# Key Role of Hyaluronan Metabolism for the Development of Brain Metastases in Triple-Negative Breast Cancer

**DOI:** 10.3390/cells11203275

**Published:** 2022-10-18

**Authors:** Fabienne Hamester, Christine Stürken, Karen Legler, Kathrin Eylmann, Katrin Möller, Maila Roßberg, Christian Gorzelanny, Alexander T. Bauer, Sabine Windhorst, Barbara Schmalfeldt, Elena Laakmann, Volkmar Müller, Isabell Witzel, Leticia Oliveira-Ferrer

**Affiliations:** 1 Department of Gynecology, University Medical Center Hamburg-Eppendorf, Martinistrasse 52, 20246 Hamburg, Germany; 2 Department of Anatomy and Experimental Morphology, University Medical Center Hamburg-Eppendorf, Martinistrasse 52, 20246 Hamburg, Germany; 3 MSH Medical School Hamburg, Faculty of Medicine, Medical University, 20251 Hamburg, Germany; 4 Department of Dermatology and Venerology, University Medical Center Hamburg-Eppendorf, Martinistrasse 52, 20246 Hamburg, Germany; 5 Department of Biochemistry and Signal Transduction, University Medical Center Hamburg-Eppendorf, Martinistrasse 52, 20246 Hamburg, Germany

**Keywords:** breast cancer brain metastases, blood-brain barrier, pericellular Hyaluronan-coat, adhesion, invasion

## Abstract

Breast cancer (BC) is the second-most common cause of brain metastases (BM) and BCBM patients have a reduced quality of life and a poor prognosis. Hyaluronan (HA), and in particular the hyaluronidase Hyal-1, has been already linked to the development of BCBM, and therefore presents an interesting opportunity to develop new effective therapeutic options. HA metabolism was further discovered by the CRISPR/Cas9-mediated knockout of HYAL1 and the shRNA-mediated down-regulation of HA-receptor CD44 in the brain-seeking triple-negative breast cancer (TNBC) cell line MDA-MB-231-BR. Therefore, the impact of Hyal-1 on adhesion, disruption, and invasion through the brain endothelium, both in vitro and in vivo, was studied. Our analysis points out a key role of Hyal-1 and low-molecular-weight HA (LMW-HA) in the formation of a pericellular HA-coat in BC cells, which in turn promotes tumor cell adhesion, disruption, and migration through the brain endothelium in vitro as well as the extent of BM in vivo. CD44 knockdown in MDA-MB-231-BR significantly reduced the pericellular HA-coat on these cells, and, consequently, tumor cell adhesion and invasion through the brain endothelium. Thus, the interaction between Hyal-1-generated LMW-HA fragments and the HA-receptor CD44 might represent a potential target for future therapeutic options in BC patients with a high risk of cerebral metastases formation.

## 1. Introduction

A significant portion, 10–30%, of all metastatic breast cancer (BC) patients develop brain metastases (BM) [1,2]. In contrast with other distant metastatic lesions, BM dramatically reduce the quality of life and prognosis of BC patients [3]. Here, neurological deficits, including both cognitive and sensory dysfunction [4], are often observed, and the average life expectancy after breast cancer brain metastases (BCBM) diagnosis is 2 to 25 months [5,6,7]. In most cases, BM formation is preceded by metastases in other organs, such as the lung, liver, or bones [1]. Therapy options in the metastatic setting include surgical resection of metastases, whole-brain radiotherapy, stereotactic radiation, and targeted therapies. However, the chances of complete tumor reduction are frequently limited [5,8,9], in part due to the limited penetration of therapeutic compounds beyond the blood-brain barrier (BBB) [10]. The BBB represents a highly organized physiological barrier, composed of endothelial cells, pericytes, and astrocytic endfeet, and is characterized by a selective permeability, with the aim to protect the central nervous system (CNS) and to maintain brain homeostasis. In this context, tumor cells require specific molecular characteristics to overcome the BBB and establish brain metastatic lesions [11,12,13]. In order to develop successful therapy options for BCBM patients, but also in order to predict a predisposition of primary BC patients to develop BM, we must improve our knowledge of the molecular characteristics required for tumor cells to interact and successfully pass the BBB.

In BC, the molecular subtype of the primary tumor is strongly associated with BM prevalence. Previous studies have shown that patients of the triple-negative breast cancer (TNBC) and HER2+ subtype have a higher risk of developing BM [14,15,16,17]. Further, the molecular subtype is not only decisive for the incidence, but also impacts how fast BMs progress, and predicts the survival rates. Patients from the luminal A group showed the highest median survival, whereas patients from the TNBC group showed the lowest median survival (luminal A: 23.1, luminal B: 15.0, HER2: 12.5, and TNBC: 6.4 months, respectively) [18,19].

Several studies have shown an association between the BC molecular subtype and the hyaluronan (HA) metabolism rate. HA, a linear glycosaminoglycan (GAG) consisting of repeating disaccharides, is synthesized by HA synthases (HAS), a group of transmembrane proteins directly located at the plasma membrane and cleaved into smaller fragments by different hyaluronidases (HYAL) [20]. In this context, HA fragments of different molecular weights can be found: high-molecular-weight HA (HMW-HA), which is mainly present under physiological conditions, and low-molecular-weight HA (LMW-HA), which is increasingly secreted under pathological conditions [21,22]. While HMW-HA exerts anti-angiogenic, anti-inflammatory, and anti-proliferative functions, LMW-HA activates signaling cascades that promote angiogenesis, immune cell influx proliferation, and migration [22]. HA molecules can be found as a component of the extracellular matrix, bound to the cell surface, or freely available after being released by many cell types. In recent years, the function of membrane-bound HA has been increasingly studied. Here, HA forms a glycocalyx together with other GAGs, which supports the cell–cell binding. This outer HA layer, which is regarded as a pericellular HA-coat, can be generated via binding to the HA synthases during synthesis and extrusion, or through the interaction to its cognate receptor CD44 or the receptor for hyaluronan-mediated motility (RHAMM), potentially resulting in the activation of a variety of intracellular signaling pathways [23].

Interestingly, TNBC cells display endogenously higher HA levels compared to hormone-receptor-positive ones [24], thereby attributing a role to HA during tumor formation and progression. Further, the expression level of enzymes associated with HA metabolism is often increased in tumor cells compared to healthy tissue. Li et al. have shown an association between an increased Has-2 level and BC tumor progression [25]. We have previously reported the impact of HA metabolism and BM development in BC. Here, an increased Has-2 and especially Hyal-1 level in BC patients is associated with a significantly higher risk of BM formation [26]. In the present study, we have analyzed the impact of (1) the HA-tumor coat in BC cells and (2) the HYAL1 depletion in TNBC cells on the tumor cell interaction with the BBB in vitro, and on the development of BM in vivo. 

## 2. Materials and Methods

### 2.1. Cell Lines

All used cell lines were purchased and cultured as previously described by Hamester et al. [27]. 

### 2.2. CRISPR/Cas9-HYAL1 Knockout in MDA-MB-231-BR

CRISPR/Cas9-HYAL1 knockout (KO) in MDA-MB-231-BR cells was performed according to the plasmid-based procedure described by Ran et al. [28]. Two guide sequences, both located on exon 2 of the HYAL1 gene, were selected with the provided CRISPR guide RNA design tool guide scan. Guide sequences and primers used for sgRNA oligo insert construction can be found in the supplementary materials and methods file. The sgRNA oligos were cloned into pSpCas9(BB)-2A-GFP plasmid (Addgene (Watertown, MA, USA) plasmid ID: 48138). MDA-MB-231-BR cells were transfected with HYAL1-specific CRISPR/Cas9 plasmid, as well as empty vector (Ctrl), by using Lipofectamine^®^ LTX Reagent (#15338100, Thermo Fisher Scientific, Waltham, MA, USA). Single-cell-colonies were assessed using the FACS Sorting method, and HYAL1 KO was checked via sequencing of genomic DNA. The primer for sequencing can also be found in the supplementary materials and methods file. 

### 2.3. HYAL1 and HAS2 Overexpression in MCF-7

The full HAS2 and HYAL1 cDNA sequences were obtained from a commercially available vector (pCR-Blunt II-TOPO-HAS2 Plasmid, Source Bioscience, Nottingham, UK; IRCMp5012E0230D, and pCMV-Sport6-HYAL1 Plasmid; Source Bioscience, Nottingham, UK; IRATp970C1157D) and cloned into LeGO-iC2-Puro+ Plasmid and LeGO-iG2-Neo+ Plasmid, respectively (a kind gift from AG Fehse, Center for Oncology, Department of Stem Cell Transplantation, UKE, Hamburg, Germany). After lentiviral production in HEK293T cells, MCF-7 cells were transduced. The corresponding empty vector was taken as a negative control. 

### 2.4. CD44 Knockdown (KD) in MDA-MB-231-BR

CD44 knockdown in MDA-MB-231-BR cell line was generated by lentiviral transduction (lentiviral vectors were a kind gift from Daniel Wicklein and Kristoffer Riecken from the Department of Anatomy and Experimental Morphology and the Department of Stem Cell Transplantation, at the UKE, Hamburg, Germany, respectively) using vectors containing shRNA-sequences which targeted specific regions of the CD44 mRNA sequence (pLVX-shCD44 (1 CD44 IV)). Similarly, a control cell line was established using a scramble shRNA sequence (pLVX-shScrambled). 

### 2.5. Treatment with Hyaluronidase

All cell lines were incubated with 10 U/mL Hyaluronidase from bovine testes (#H3506-100MG, Sigma Aldrich, St. Louis, MI, USA) in serum-reduced media (3% (*v*/*v*) FBS (#F9665, Thermo Fisher Scientific Waltham, MA, USA)) overnight, and functional assays were subsequently performed.

### 2.6. HA-Binding Protein (HABP) Staining and Immunohistochemistry 

For HABP histochemistry, cells were first embedded in paraffin (supplementary materials and methods), and a staining procedure was performed as previously described [29]. Briefly, antigen retrieval was performed using the 1× Target Retrieval Buffer (#S1699, Dako, Agilent Technologies Inc., Santa Clara, CA, USA) in a water bath at 60–65 °C overnight, and, subsequently, slides were blocked with a 1% (*w*/*v*) BSA/TBS (bovine serum albumin (BSA, #8076.1, Carl Roth, Karlsruhe, Germany)/Tris-Buffered Saline (TBS)) solution for 30 min at room temperature (RT), and incubated with an HABP (#385911, Merck Millipore, Burlington, MA, USA) solution (1:75, c = 6.6 g/mL) for 90 min at room temperature. The HA-binding protein was visualized using the ABC-AP and the permanent AP Red Kit (#AK-5000, Vector Laboratories). For the luciferase staining, antigen retrieval was performed using 1× Citrate-buffer (pH 6) in a steamer (100 °C, 20 min), and subsequent incubation with Luciferase primary antibody (#ab181640, Abcam, Cambridge, UK) was performed (1:500 in Antibody Diluent (#S0809, Dako, Agilent Technologies Inc., Santa Clara, CA, USA, overnight at 4 °C), followed by incubation with a biotinylated anti-Goat IgG secondary antibody (#BA-9500, Vector Laboratories, Burlingame, CA, USA; 10 mL TBS, 50 µL Normal Horse Serum (#S-2000-20, Vector Laboratories) and 100 µL anti-Goat IgG), for 30 min at RT). Antibody visualization and nuclei counterstaining were performed as described above.

The HABP fluorescence staining was performed on cells previously seeded on coverslips. After cell fixation (20 min) with a 3.7% formaldehyde solution (#7398.1, Carl Roth), a blocking step was performed (1 h with a 1% BSA/PBS solution) followed by incubation with the biotinylated hyaluronic acid-binding protein solution (HABP, #385911, Merck Millipore, Burlington, MA, USA; 1:75 in 1% BSA/PBS, 45 min, RT). Cells were then incubated with a secondary antibody solution (Streptavidin-FITC, #405202, BioLegend, San Diego, CA, USA; 1:500 in 1% BSA/PBS, 60 min, RT) and subsequently with DAPI (#9542, Sigma Aldrich, St. Louis, MI, USA; 1:1000 in PBS [#D8537, Merck Millipore, Burlington, MA, USA], 10 min, RT, dark). After washing, coverslips were transferred to glass slides and mounted with Fluoromount-G™ (#00-4958-02, Invitrogen, Waltham, MA, USA).

### 2.7. F-Actin Fluorescence Staining

For staining of cytoskeletal F-actin, 5 × 10^4^ cells were seeded on sterile coverslips into 24-well plates and cultured for 72 h. Cells were washed and fixed as described above. After blocking for 1 h at room temperature with 1% BSA/PBS, cells were stained with phalloidin (#ab235138, Abcam, 1:2000 in PBS (#D8537, Merck)) for 30 min at RT, and finally cover-slipped with Mounting Medium for Fluorescence with DAPI (#H-1200-10, Vector Laboratories). Slides were stored at −20 °C in the dark and imaged with a fluorescence microscope (Olympus, Tokyo, Japan; IXplore Live-System).

### 2.8. Western Blot

Western blot was performed as previously described [29]. The following antibodies were used: β-Actin (#sc-47778, Santa Cruz Biotechnology (SCB)), β-Catenin (#8480, Cell Signaling Technology (CST)), CD44 (#sc-7297, SCB), HSC-70 (#sc-7298, SCB), RHAMM (#sc-515221, SCB), Slug (#5741, CST), Snail (#3879, CST), TCF8/ZEB1 (#3396, CST), Vimentin (#5741, CST), ZO-1 (#8193, CST), Anti-mouse (#sc-2055, SCB), and Anti-rabbit (#sc-2357, SCB). Detection was performed using the Westar Nova 2.0 Chemiluminescent Substrate for Western blotting Kit (#XLS071,0250, Cyanagen Srl., Bologna, Italy). Densitometric evaluation of the protein bands was performed using the GS-800 Calibrated Densitometer and Quantity-One-4.6.3 software from Bio-Rad, Hercules, CA, USA. Two independent western blot analyses, as well as subsequent quantification and statistical analysis, have been performed for all experiments shown in this study. Figures display one representative Western blot image.

### 2.9. Enzyme-Linked Immunosorbent Assay (ELISA)

In order to analyze the amount of HA in cell culture supernatants, 1 × 10^6^ cells were seeded into T75-flasks and cultured to a confluence of 80%. Cells were washed 2 times with PBS (#D8537, Merck Millipore, Burlington, MA, USA), and cultured with serum-reduced media (3% FBS) for another 48 h. Cell culture media were collected and centrifuged (10 min, 12,000 rpm, RT). The resulting supernatant was used for HA-ELISA (ELISA-like Assay for HA/Hyaluronan, #AMS.CSR-HA-96KIT, Amsbio, Abingdon, UK). ELISA was performed according to the manufacturer’s protocol. The amount of HA was normalized to the cell count at the time of taking supernatants. 

### 2.10. HYAL1-Activity Gel-Assay

Tumor cells were seeded in 6-well-plates and cultivated until they reached 80% confluency. Isolated protein lysates were incubated with HMW-HA in a Hyal-1 working buffer (1% Triton X-100 (#9036-19-5, Merck Millipore, Burlington, MA, USA), 150 mM NaCl (#7647-14-5, Merck Millipore, Burlington, MA, USA), 100 mM formic acid (#,64-18-6, Merck Millipore, Burlington, MA, USA, pH 3.7) for 14 h at 37 °C in a heating block. A 0.5 cm thick 1% agarose (#15581044, Thermo Fisher Scientific, Waltham, MA, USA) gel in 1× TAE buffer (2 M Trizma^®^ base (#77-86-1, Merck Millipore, Burlington, MA, USA), 1 M pure acetic acid (#64-19-7, Merck Millipore, Burlington, MA, USA), 0.05 M EDTA (#60-00-4, Merck Millipore, Burlington, MA, USA, pH 8.3), was prepared and pre-runed for 6 h at 80 V. All samples were subsequently electrophoresed for 2 h at 100 V (10 µL sample + 2 µL 6× loading Dye (#R0611, Thermo Fisher Scientific, Waltham, MA, USA)).The gel was equilibrated in a box with 30% ethanol in water by shaking for 1 h at RT. Ethanol was replaced, and the gel was incubated in Stains-All working solution (500 µL 1× Stains-All (#7423-31-6, Merck Millipore, Burlington, MA, USA, 200 mL 30% ethanol, overnight, dark, RT). The gel was de-stained with 30% ethanol in water (overnight, dark, RT).

### 2.11. Quantitative Real-Time PCR

RNA extraction and real-time qPCR was conducted as previously described [27]. All used primers are listed in the supplementary materials and methods section.

### 2.12. Flow Cytometry

Next, 2 × 10^5^ cells were stained with biotinylated hyaluronic acid binding protein (HABP, #385911, Merck Millipore, Burlington, MA, USA; 1:75 in 1% BSA/PBS, 30 min, 4 °C) or CD44-AF488 (#338829, clone BJ18, BioLegend, San Diego, CA, USA; 1:200 in 1% BSA/PBS, for 20 min at 4 °C), and, after a washing step, were incubated with a secondary antibody (Streptavidin-FITC, #405202, BioLegend, San Diego, CA, USA; 1:500 in 1% BSA/PBS) for 20 min at 4 °C. Labeled cells were measured with FACSCalibur (Becton Dickinson, Franklin Lakes, NJ, USA). The analysis was performed using FlowJoV10 Software (Version v10.8.1). At least three independent flow cytometry analyses, as well as subsequent quantification and statistical analysis, have been performed for all experiments shown in this study. If this was not the case for individual experiments, it was mentioned separately in the figure legends. Figures display one image from a representative flow cytometry experiment.

### 2.13. Static Cell Adhesion Assay

Static cell adhesion assay to analyze the adhesion ability of different tumor cells to human brain endothelial cells was performed as previously described [27]. 

### 2.14. Electrical Cell-Substrate Impedance Sensing (ECIS)

The effect of different tumor cells on the BBB integrity was examined by an electrical cell-substrate impedance sensing system (ECIS), as previously described [27]. Results represent three to five replicates from each cell type or donor. 

### 2.15. Transwell Invasion Assay

Transwell invasion assay was used to analyze the potential of different tumor cells to invade through a brain endothelial cell monolayer. The assay was performed as previously described by Hamester et al. [27].

### 2.16. Migration Assay

Cells (2 × 10^5^ per well) were seeded in six-well plates and cultured until confluence. An artificial wound was created with the help of a pipette tip in each well. The migration potential of the cells was determined by analyzing the wound area at different time points (0, 10, and 24 h) with the ImageJ Wound Healing Tool (Version 1.52t, Wayne Rasband, National Institute of Health, Bethesda, MA, USA). We have assessed the change in the wound width over time for each individual well. This width is defined as the average distance between the two margins of the scratch. Precisely, for each individual well wound, widths at the same position were measured at different time points and calculated as a percentage of the value at time point 0 h (100%).

### 2.17. Intracardiac Metastasis Mouse Model 

An intracardiac mouse model to analyze development of brain metastases was conducted as previously described [27]. In contrast to the method described so far, anesthetized mice were imaged from both lateral and ventral views approximately 10–15 min after intraperitoneal injection of D-luciferin, using the IVIS^®^ Spectrum in vivo imaging system (PerkinElmer, Waltham, MA, USA). The animal experiments were approved by the Authority for Social Affairs, Family, Health, and Consumer Protection of the Free and Hanseatic City of Hamburg, through application N005/2020.

### 2.18. Statistics

Statistical analysis was conducted using SPSS software Version 25 (IBM SPSS Statistics, Armonk, NY, USA). For all functional assays, cells were plated in triplicates and each experiment was performed three times (*n* = 3). Statistical significance was determined using unpaired two-tailed Student’s *t*-tests. The assumption of homogeneity of variance was tested using Levene’s Test of Equality of Variances (*p* > 0.05). Results are given as mean +/− s.d. All *p*-values less than 0.05 were considered statistically significant. 

## 3. Results

### 3.1. HA Metabolism and HA Pericellular Coat in BC Cell Lines Corresponding to Different Molecular Subtype

**Figure 1:**
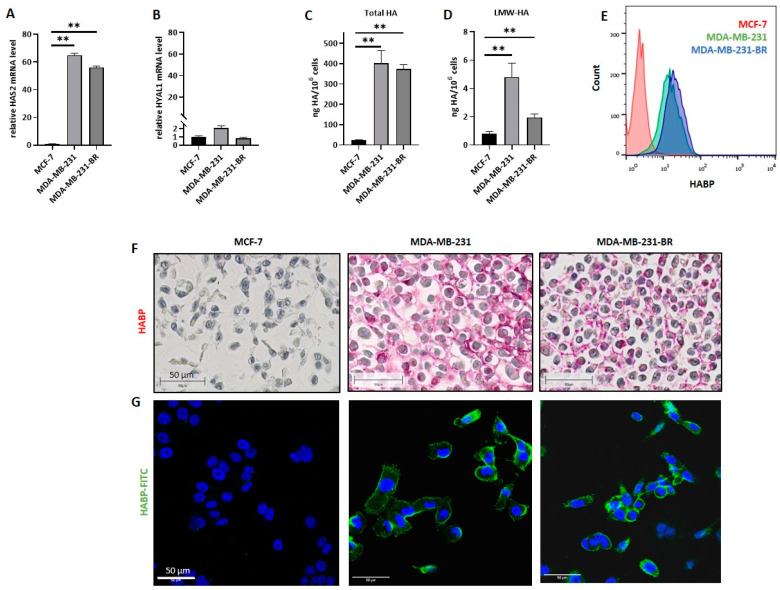
Hyaluronan metabolism in breast cancer (BC) cell lines of different molecular subtypes. The relative mRNA expression level of (**A**) hyaluronan synthase 2 (HAS2) and (**B**) hyaluronidase 1 (HYAL1) was measured by qRT-PCR. Values are normalized to corresponding GAPDH expression (*n* = 3). (**C**) Bar graph displaying ELISA results on secreted hyaluronan (HA) levels (ng/10^6^ cells) of MCF-7, MDA-MB-231, and MDA-MB-231-BR. Cell supernatant was collected and used for HA-ELISA; one representative experiment with three replicates out of three experiments is shown (*n* = 3). (**D**) Levels of secreted low-molecular-weight HA (LMW-HA) (ng/10^6^ cells) of MCF-7, MDA-MB-231, and MDA-MB-231-BR measured by HA-ELISA; one representative experiment with duplicates out of three experiments is shown (*n* = 3). (**E**) Histogram showing the quantified amount of surface HA, measured via flow cytometry using biotinylated HA binding protein and FITC-conjugated Streptavidin. One representative experiment is shown (*n* = 3). (**F**) Immunohistochemical staining of HA (pink) using biotinylated HA binding protein of all three cell lines. Representative picture out of 3. Scale bars represent 50 µm. (**G**) Immunofluorescence staining of nuclei (DAPI, blue) and HA (FITC, green) using biotinylated HA binding protein and FITC-conjugated Streptavidin for all three cell lines. Representative picture out of 3. Scale bars represent 50 µm. Statistical significance was determined using unpaired two-tailed Student’s *t*-tests. The assumption of homogeneity of variance was tested using Levene’s Test of Equality of Variances (*p* > 0.05). Values are means +/− s.d., ** *p* < 0.005.

Firstly, the mRNA expression level of two major HA-related enzymes, the synthase Has-2 and the hyaluronidase Hyal-1, which have been previously associated with BM formation in BC patients, were examined in three BC cell lines corresponding to two different molecular subtypes, namely MCF-7 (luminal), MDA-MB-231 (TNBC), and MDA-MB-231-BR (brain-seeking TNBC) (Figure 1A,B). Here, both TNBC cell lines showed significantly higher HAS2 mRNA levels compared to the hormone receptor-positive cell line, whereas the mRNA expression level of HYAL1 was low overall, and differed only slightly in all three cell lines. In the second step, total and surface-bound HA levels were quantified. Here, the amount of secreted HA per 10^6^ cells was detected and quantified via HA-ELISA (Figure 1C,D). Both MDA-MB-231 and MDA-MB-231-BR synthesized and secreted significantly more total HA and small HA fragments (LMW-HA) compared to MCF-7 cells. Quantification of the cell surface-bound HA using flow cytometry corroborated high levels of membrane-bound HA in both TNBC cell lines, while for MCF-7, only small amounts of HA could be detected (Figure 1E). The brain-seeking subline showed a slightly higher HA surface level than the parental cell line (geomean values: MCF-7 = 2.58, MDA-MB-231 = 13.9, and MDA-MB-231-BR = 19.4), suggesting a more compact pericellular HA-coat. In order to visualize the secreted HA-fragments and the potential pericellular HA-coat, we stained all three cell lines using a biotinylated HA-binding protein via histochemical and fluorescence staining (Figure 1F,G). Only the TNBC cell lines MDA-MB-231 and MDA-MB-231-BR showed a positive HABP staining, whereas MCF-7 did not. Furthermore, we observed a different HA staining pattern between the MDA-MB-231 cell line and its brain-seeking subline. While the parental cell line displayed long HA fragments corresponding to both a pericellular and extracellular HA pattern, HA was predominantly restricted to the cell membrane in MDA-MB-231-BR cells. 

### 3.2. Impact of the Tumor HA-Coat on Cell Adhesion to the Brain Endothelium

**Figure 2:**
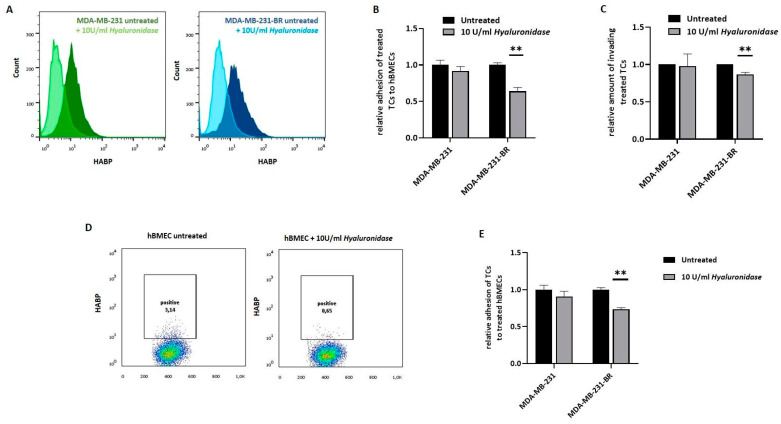
Influence of the pericellular HA-coat on BBB-related adhesion. (**A**) Histogram showing surface HA measured via flow cytometry: for both triple-negative breast cancer (TNBC) cell lines (MDA-MB-231, MDA-MB-231-BR) the histogram shows the HA level in the untreated situation (dark color) as well as after treatment with 10 U/mL *hyaluronidases* (light color). One representative experiment is shown (*n* = 2). (**B**) Adhesion of tumor cells (TCs, untreated) and previously treated TCs (10 U/mL *hyaluronidase*, overnight) to human primary brain endothelial cells (hBMECs) analyzed under static conditions. The relative amount (to untreated control = 1) of adhesive TCs is shown (one representative experiment with six replicates out of four experiments is shown (*n* = 4)). (**C**) Invasion potential of TCs (untreated) and previously treated TCs (10 U/mL *hyaluronidase*, overnight) through a brain endothelial cell monolayer was measured in a transwell assay. The relative amount (to untreated control = 1) of invasive TCs is shown (*n* = 3). (**D**) Dot plots showing the quantified amount of surface HA on hBMECs in the untreated situation as well as after treatment with *hyaluronidase* (10 U/mL), measured via flow cytometry. One representative experiment is shown (*n* = 2). (**E**) Adhesion of TCs to hBMECs (untreated) or previously treated hBMECs (10 U/mL *hyaluronidase*, overnight) was analyzed under static conditions. The relative amount (to untreated control = 1) of adhesive TCs is shown (one representative experiment with six replicates out of four experiments is shown (*n* = 4)). Statistical significance was determined using unpaired two-tailed Student’s *t*-tests. The assumption of homogeneity of variance was tested using Levene’s Test of Equality of Variances (*p* > 0.05). Values are means +/− s.d., ** *p* < 0.005.

Next, we were interested in whether the pericellular HA-coat influences the tumor cell interaction with the brain endothelium. Here, we used primary human brain microvascular endothelial cells (hBMEC) instead of a brain endothelial cell line, as precisely these primary cells have been described to preserve the native cell morphology and maintain many key features of the BBB in vitro [30,31,32]. The pre-existing HA-coat was enzymatically removed from the TNBC cell surfaces via exogenous hyaluronidase (10 U/mL, overnight) treatment. A mixture of different kinds of hyaluronidases, a group of HA metabolic enzymes that cleaves HA in small fragments of up to 200 Da, was used. The effect of the enzymatic reaction on the HA-coat was first confirmed via flow cytometry for both TNBC cell lines (Figure 2A). As expected, treatment with hyaluronidase resulted in a strong reduction in the surface HA level for MDA-MB-231 and MDA-MB-231-BR cells. Subsequently, the impact of the HA-coat on the tumor cell characteristics was investigated. The effect of the hyaluronidase treatment on tumor cell adhesion to brain endothelial cells is displayed in Figure 2B, where adhesion values were normalized to the untreated situation for each cell line. Interestingly, the reduction in the HA-coat showed a significant effect only on MDA-MB-231-BR cells, leading to a significantly decreased (36% less adhesion) tumor cell adhesion to hBMECs (*p* = 0.003). As for the adhesion, the role of the pericellular HA-coat on tumor cells during invasion through the brain endothelium was examined (Figure 2C). Tumor cells were allowed to invade through the endothelium for 24 h, and invasive potential, as assessed by the transwell assay, without previous treatment was set as 1 for each cell line. While the HA-coat removal in the invasive MDA-MB-231 cell line did not affect tumor cell invasion, it resulted in a significant decrease in the invasive potential in the brain-seeking subline. This finding let us assume that, despite an approximately equal level of pericellular HA-coat in MDA-MB-231 and MDA-MB-231-BR cells, tumor cell adhesion and invasion through the brain endothelial might be more dependent on this glycan layer for the brain-metastatic subline.

Besides the tumor cell HA-coat, the endothelium has been described to actively build a pericellular HA layer as well. In order to investigate whether the HA layer on the endothelial cell surface also affects tumor cell adhesion, we performed a hyaluronidase treatment in hBMECs, similar to the previously described for the cell lines. The HA-coat after treatment was quantified via flow cytometry (Figure 2D). Here, although the HA levels in untreated cells were low, the hyaluronidase treatment led to a measurable reduction in the HA-positive cells, from 3.1% to 0.65%. Interestingly, this slight HA-coat reduction still affected the MDA-MB-231-BR interaction with the endothelial cell monolayer, resulting in a significantly decreased (27% less adhesion) tumor cell adhesion. In contrast, the hyaluronidase treatment on endothelial cells did not affect the tumor cell adhesion of MDA-MB-231 cells (Figure 2E).

### 3.3. HYAL1 Knockout in MDA-MB-231-BR Cells Reduces the Pericellular HA-Coat

**Figure 3:**
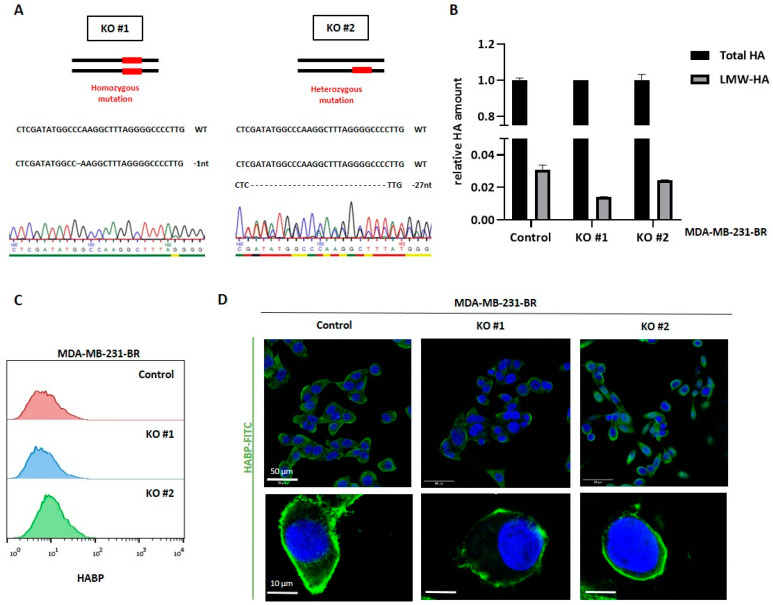
Generation of HYAL1 knockout (KO) in MDA-MB-231-BR and characterization regarding their HA metabolism. (**A**) Two types of HYLA1 mutations generated by CRISPR/Cas9 in MDA-MB-231-BR cells were identified after Sanger sequencing in the corresponding sequencing chromatogram. Mutation features shown here include a homozygous single-base deletion in clone KO #1 (indicated as -1nt) and a larger deletion in only one allele of clone KO #2 (-27nt). (**B**) Bar graph displaying representative HA-ELISA results on secreted HA levels (one representative experiment with duplicates out of three experiments is shown (*n* = 3)). The amount of LMW-HA is presented as a ratio of total HA (set as 1). (**C**) Multilayer histogram showing the quantified amount of surface HA measured via flow cytometry, using biotinylated HA binding protein and FITC-conjugated Streptavidin. One representative experiment is shown (*n* = 3). (**D**) Immunofluorescence staining of nuclei (DAPI, blue) and HA (FITC, green) using biotinylated HABP and FITC-conjugated Streptavidin. Scale bars represent 50 µm in the upper panel and 10 µm in the lower panel. Statistical significance was determined using unpaired two-tailed Student’s *t*-tests. The assumption of homogeneity of variance was tested using Levene’s Test of Equality of Variances (*p* > 0.05). Values are means +/− s.d.

As demonstrated in the previous sections, the metastatic BC cell lines, MDA-MB-231 and MDA-MB-231-BR, exhibit a pronounced HA metabolism and are characterized by a robust pericellular HA-coat, playing a key role during early cell adhesion and invasion, particularly in the brain-seeking cell line. On the other hand, our previously published study on HA and BCBM [26] pointed out a strong association between the expression level of the hyaluronidase Hyal-1 in the primary tumor and the development of BM in BC patients. These data together suggest a key role of HA and precisely of the LMW-HA fragments, cleaved by Hyal-1, during BM formation. In order to investigate the specific role of Hyal-1 in BCBM, HYAL1 knockout (KO) was generated using the CRISPR/Cas9 method in MDA-MB-231-BR cells. Single-cell clones were isolated by FACS sorting, and two clones were further characterized regarding the resulting genomic alterations, as well as phenotypically. Clone KO #1, a homozygous one, showed a deletion of one nucleotide in both alleles, whereas the heterozygous clone KO #2 exhibits one wild-type allele and a deletion of 27 nucleotides in the second allele (Figure 3A), which might, rather, represent a HYAL1 knockdown subline. 

Both clones were further characterized regarding their HA metabolism and their cellular properties. No significant differences in the secreted amount of total HA between KO #1 or KO #2 and the control cells could be detected by HA-ELISA (Figure 3B, black rows). However, the amount of LMW-HA is slightly reduced in both HYAL1 KO clones, resulting in a relatively low amount of LMW-HA compared to the respective total HA amount, as shown in Figure 3B (grey rows). This trend was more pronounced in KO #1, although no statistical significance could be found. Regarding the total amount of surface-bound HA, the FACS analysis showed a slightly higher expression of pericellular HA on KO #2 compared to the control, whereas KO #1 behaved similarly to the control in this regard (Figure 3C). Here, one possible explanation might be the counter-regulation of other HA-related enzymes as a consequence of the HYAL1 KO. Indeed, the qRT-PCR analysis showed a significant up-regulation of hyaluronidase 2 (HYAL2) in both clones, and, in line with the FACS results, a strong up-regulation of synthase 2 (HAS2) in the KO #2 subline (Supplementary Figure S1A). 

The immunofluorescence staining showed more precisely that the cell surface-bound amount of HA, i.e., the pericellular HA coat, is especially reduced in both HYAL1 KO clones (Figure 3D). This finding suggests that LMW-HA fragments, which are less predominant in HYAL1 KO clones, are important for the formation of the pericellular HA-coat. In the KO clones, this layer is still present on the surface of the cells, meaning that quantitative measurement by FACS did not yield a difference, but the HA-coat was macroscopically thinner due to the lower amount of LMW-HA fragments. 

### 3.4. HYAL1 KO in MDA-MB-231-BR Cells Alters the Cellular Phenotype and Results in the Reorganization of the Actin Cytoskeleton

**Figure 4:**
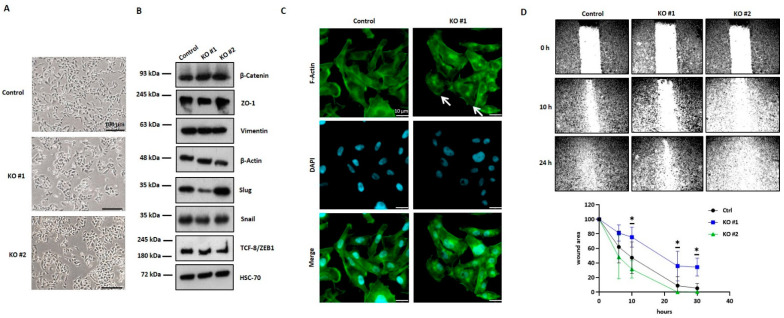
Phenotypic changes in HYLA1 KO cells and their influence on mesenchymal–epithelial transition (MET) and migration. (**A**) Representative pictures of HYAL1 KO MDA-MB-231-BR cells and the corresponding control. Scale bars represent 100 µm. (**B**) Representative Western blot analysis (*n* = 2) of protein lysates (20 µg) of MDA-MB-231-BR control, HYAL1 KO #1, and HYAL1 KO #2 showing expression of different MET markers. HSC-70 (70 kDa) and β-Actin (45 kDa) were used as loading controls. (**C**) Immunofluorescence staining of F-actin (Alexa-fluor568 conjugated phalloidin). Scale bars represent 10 µm. (**D**, upper panel) Representative pictures showing migration ability of MDA-MB-231-BR control, HYAL1 KO #1, and HYAL1 KO #2 cells at the time of wound scratching (0 h), 10 h, and 24 h post-scratching. Magnification 5× (**D**, lower panel). Graphical representation of the wound closure in % of the existing wounding area (*n* = 3). Statistical significance was determined using unpaired two-tailed Student’s *t*-tests. The assumption of homogeneity of variance was tested using Levene’s Test of Equality of Variances (*p* > 0.05). Values are means +/− s.d. * *p* < 0.05.

In addition to the HA-dependent changes in HYAL1 KO cells, we observed morphological alterations, especially in clone KO #1 (Figure 4A). These cells grew in small cell clusters and displayed a marked epithelial cell shape, in contrast to the mesenchymal-like morphology observed in the control cells. A mesenchymal–epithelial transition (MET) in these cells might explain this morphological change. In order to explore this possibility, the expression level of different MET markers was analyzed using Western blot (Figure 4B). For HYAL1 KO, a slightly increased expression of the cell junction protein ZO-1 (220 kDa) was detected, supporting the more epithelial-like phenotype described previously. In addition, HYAL1 KO resulted in reduced expression of the transcription factors Slug (30 kDa), Snail (29 kDa), and TCF-8/ZEB1 (200 kDa), all three makers promoting the mesenchymal phenotype, whereas no differences in the expression level could be detected for β-Catenin (92 kDa) and Vimentin (57 kDa). However, all of these altered expression levels of MET markers were quantified (Supplementary Figure S1B–G), but were not always equally pronounced in both clones, which is why a Hyal-1-specific effect cannot be assumed here.

Next, we analyzed the impact of HYAL1 KO on the cell cytoskeleton. Figure 4C shows the immunofluorescence staining for F-actin using phalloidin red. While for control cells, the typical mesenchymal-like cell shape was detected, KO #1 showed a more epithelial phenotype and the formation of stress fibers (white arrow). Based on these findings, the cell migration potential was analyzed by the scratch assay. Representative pictures of migrating cells 10 and 24 h after wound scratching are shown in Figure 4D (upper panel). While the wounding area of the control cells and that of the KO #2 cells was almost closed after 24 h, KO #1 cells migrated much more slowly, as shown in the quantification graphic (Figure 4D, lower panel).

### 3.5. HYAL1 KO in MDA-MB-231-BR Cells Modulates Brain-Metastatic Properties In Vitro

**Figure 5:**
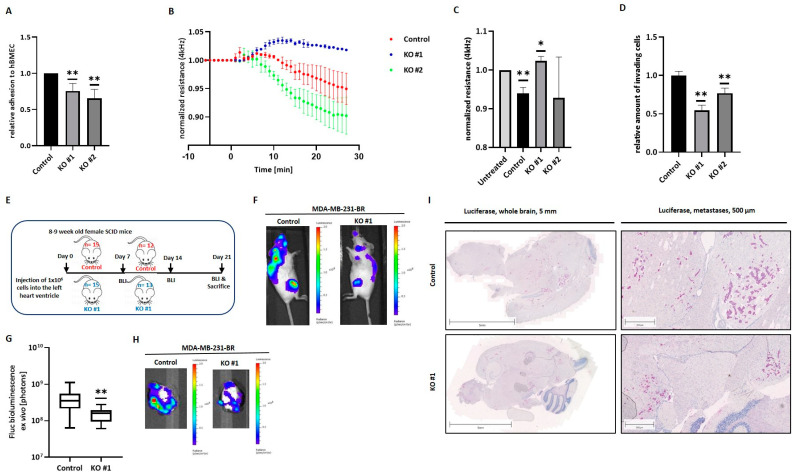
Effect of HYAL1 KO on the interaction between MDA-MB-231-BR cells and the brain endothelium in vitro and BM formation in vivo. (**A**) Tumor cell (MDA-MB-231-BR control, HYAL1 KO #1, HYAL1 KO #2) adhesion to primary human brain endothelial cells (hBMECs) was analyzed under static conditions. The relative amount (to control cells = 1) of adhesive cells is shown (*n* = 5). (**B**) Electrical cell–substrate impedance sensing (ECIS): normalized resistance at 4 kHz (values were set as 1 prior treatment; representative experiment; *n* = 3). (**C**) Bar graphs displaying relative resistance values at 4 kHz under the influence of different tumor cells (*n* = 3). (**D**) Invasion potential of tumor cells (MDA-MB-231-BR control, HYAL1 KO #1, HYAL1 KO #2) through a brain endothelial cell monolayer measured by the Transwell assay (*n* = 3). (**E**) Schematic representation of the animal study design. A total of 15 mice/group were injected. (**F**) Representative bioluminescence images (BLI) of whole mice corresponding to each group (MDA-MB-231-BR control: *n* = 12, HYAL1 KO #1: *n* = 13) 21 days after injection. (**G**) Ex vivo BLI signals from brains of both groups (MDA-MB-231-BR control: *n* = 12, HYAL1 KO #1: *n* = 13). (**H**) Representative pictures of BLI-measured brains (MDA-MB-231-BR control: *n* = 12, HYAL1 KO #1: *n* = 13) at the final time point (21 days after injection). (**I**) Immunohistochemical staining of brain tissue Luciferase (MDA-MB-231-BR control: *n* = 12, HYAL1 KO #1: *n* = 13). Corresponding scales are indicated above the pictures. Statistical significance was determined using unpaired two-tailed Student’s *t*-tests. The assumption of homogeneity of variance was tested using Levene’s Test of Equality of Variances (*p* > 0.05). Values are means +/− s.d. * *p* < 0.05, ** *p* < 0.005.

Next, the impact of HYAL1 KO on tumor cell interaction with the brain endothelium was studied in vitro. Tumor cell adhesion to hBMECs was analyzed, and results are displayed in Figure 5A. Here, a significantly lower adhesion ability of HYAL1 KO tumor cells to the brain endothelium for both clones, KO #1 and KO #2, compared to the WT cells was detected. 

Furthermore, the effect of tumoral HYAL1 KO on the BBB integrity was examined by an electrical cell-substrate impedance sensing system (ECIS). Figure 5B shows the time course of 10 min before and 30 min after the addition of tumor cells to the endothelial cell monolayer of a representative experiment. While the control cells, as well as the KO #2 cells, steadily reduced the resistance value and thus caused the BBB opening, the resistance value remained nearly constant after the addition of KO #1. This trend could be reproduced in two further experiments, whereas the extent of clone KO #2’s impact on the resistance of the brain endothelial cell monolayer strongly differed between all three experiments. Figure 3C represents normalized average resistance values an average of 30 min after the addition of tumor cells for all experiments. As expected, a significant decrease in the resistance value and, thus, relaxation of the cell–cell contacts between the brain endothelial cells compared to the untreated situation was measured when MDA-MB-231-BR cells were added to the endothelial cell monolayer. In contrast, clone KO #1 seemed to strengthen the endothelial barrier by significantly increasing the resistance, while no significant change in the resistance values with KO #2 cells was observed (Figure 5C). 

After loosening the cell–cell contacts between brain endothelial cells, tumor cells eventually migrate through. Thus, the invasive potential of MDA-MB-231-BR and the corresponding HYAL1 KO clones was studied in an in vitro invasion assay, using hBMECs. In line with the previous data, we observed a significant reduction in tumor cell invasion in both HYAL1 KO clones compared to the control cells, with clone KO #1 showing a more pronounced effect (Figure 5D).

### 3.6. HYAL1 KO in MDA-MB-231-BR Cells Decreases the Brain-Metastatic Potential In Vivo

Further, the role of HYAL1 in TNBC cells during BM development was analyzed in vivo. For this purpose, the brain-seeking cell line MDA-MB-231-BR as control was compared with clone KO #1, as this homozygous clone showed more pronounced effects on the brain endothelium in vitro. These analyses were performed by injecting 1 × 10^6^ cells intracardially into the left ventricle of 8–9 week old female SCID mice. Although 15 mice per group were injected at the start of the experiment, several mice were removed from the experiment, either on the injection day or shortly after that, showing an evidently poor condition or strong bioluminescence signals in the lungs. Therefore, 12 and 13 mice, corresponding to the control group and the clone KO #1 group, respectively, were evaluated. Tumor cell spread was monitored weekly by bioluminescence imaging (BLI) and all mice were sacrificed 21 days after tumor cell injection (Figure 5E). Figure 5F shows representative bioluminescence images of one mouse from each test group, 21 days after injection. The general tumor burden in both mice groups injected with HYAL1 KO clones was remarkably lower than in the control group, as quantified by gating bioluminescence signals corresponding to brain and bone metastases (Figure 5F). Furthermore, we found a significantly reduced number of circulating tumor cells (CTCs) in the blood samples from mice corresponding to the HYAL1 KO group compared to the control group (Supplementary Figure S1A). Remarkably, ex vivo bioluminescence values of mouse brains indicated significantly reduced BM development in mice injected with HYAL1 KO cells in comparison with those injected with the MDA-MB-231-BR cell line (Figure 5G). Representative images from the BLI corresponding to each group are shown in Figure 5H. The subsequent histological analysis and the visualization of metastases using a specific luciferase staining of the brains are shown in Figure 5I. Here, representative pictures from the whole brain, as well as a section showing several metastases, are depicted. For the control group, numerous metastases were detected in the brains of all mice, distributed throughout the cerebrum. In some cases, metastases were also found in the cerebellum. In comparison, for mice injected with HYAL1 KO #2 cells, only 0 to 2 micrometastases could be detected in the whole brains of all mice belonging to this group. In addition to BLI signals of the brain, signals from other organs were also analyzed ex vivo (Supplementary Figure S2B–G). Remarkably, we found no significant differences in the extent of metastasis to other organs between the groups studied, indicating that HYAL1 KO might specifically affect the development of brain metastases.

### 3.7. HAS2 and HYAL1 Overexpression in MCF-7 Results in Elevated HA Metabolism Rate but Does Not Impact Tumor Cell Adhesion and Migration through the Brain Endothelium

The impact of Hyal-1 expression on tumor cell interaction with the brain endothelium should be confirmed by overexpressing HA-metabolizing enzymes. For this purpose, the luminal BC cell line MCF-7 was selected, exhibiting a very low HA metabolism, as shown in Figure 1. As MCF-7 cells endogenously express low levels of the synthase HAS2, and, in turn, show low HA (HMW-HA) production, overexpression of HYAL1 alone would presumably not lead to any measurable effect in this cell line due to the lack of substrate for the catabolic enzyme. If HMW-HA is present in the cell, HYAL1 can exert its function and cleave the HA polymers into LMW-HA fragments. Therefore, simultaneous overexpression of HAS2 and HYAL1, as well as single overexpression of cell sublines (HAS2 or HYAL1) was generated in MCF-7 cells. 

**Figure 6:**
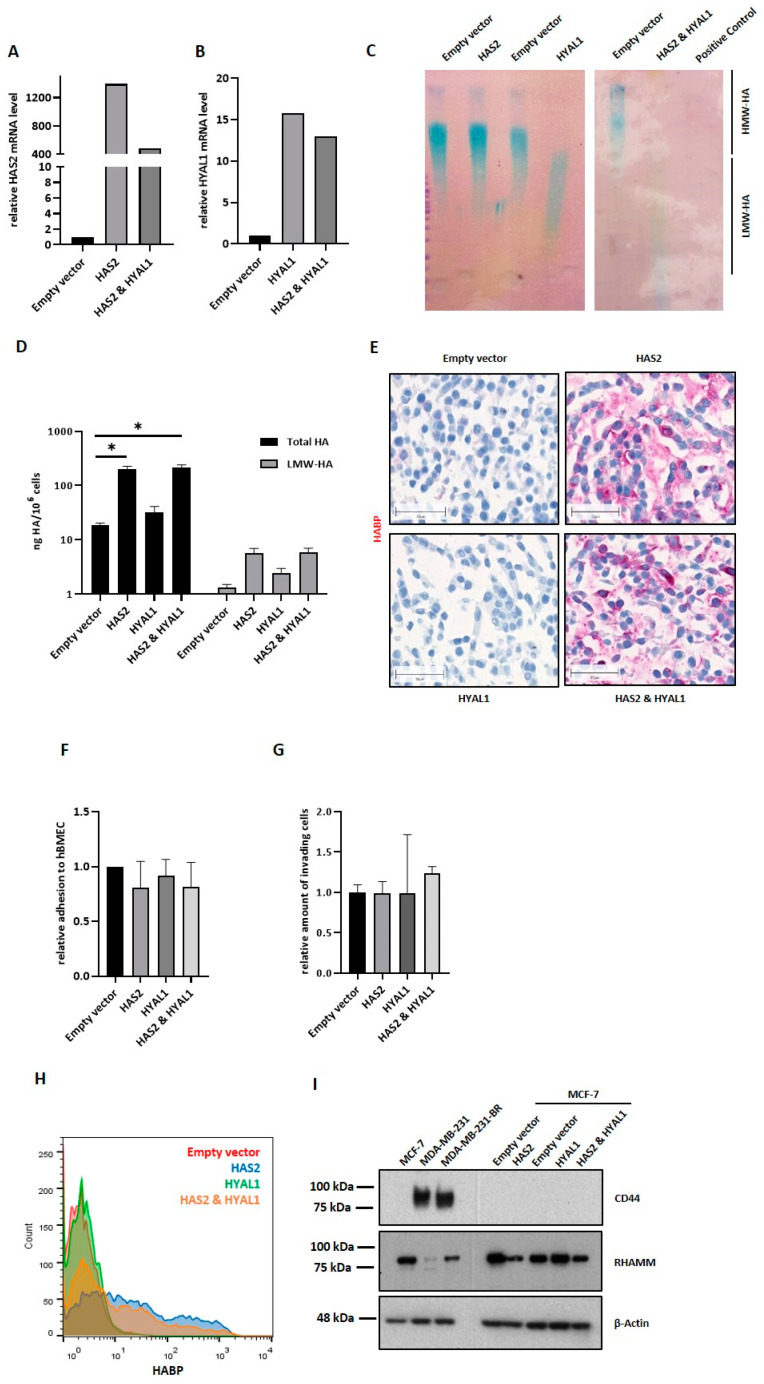
Impact of the HAS2/HYAL1 overexpression in MCF-7 cells on the interaction with the brain endothelium. Relative mRNA level of HAS2 (**A**) and HYAL1 (**B**) in MCF-7 control (empty vector), single overexpression (HAS2 or HYAL1), and double overexpression (HAS2 and HYAL1) measured by qRT-PCR. Values are normalized to corresponding GAPDH expression. (Representative; changed over time, but stable overexpression, *n* = 4). (**C**) HYAL1-activity gel-assay: HMW-HA (32 µg) was incubated for 14 h at 37 °C with 20 µg of protein lysate (RIPA (−/−)), obtained from different overexpressing MCF-7 cells and control cells. HMW-HA, incubated with 50 µL *Hyaluronidase* (1 mg/mL) served as a positive control. (**D**) Bar graph displaying HA-ELISA results on secreted total HA and LMW-HA levels (ng/10^6^ cells) of MCF-7 control (Empty vector), single overexpression (HAS2, HYAL1), and double overexpression (HAS2 and HYAL1) cells. Cell supernatant was collected and used for HA-ELISA. (**E**) Histochemical staining of cells in agar using biotinylated HA-binding protein. Scale bars represent 50 µm. (**F**) Adhesion ability of MCF-7 control (empty vector), single overexpression (HAS2, HYAL1), and double overexpressing (HAS2 and HYAL1) cells to primary human brain endothelial cells (hBMECs) analyzed under static conditions. The relative amount (to empty vector = 1) of adhesive cells is shown (*n* = 5). (**G**) Invasion potential of MCF-7 control (empty vector), single overexpression (HAS2, HYAL1), and double overexpression (HAS2 and HYAL1) through hBMECs, measured in a transwell assay (representative experiment, *n* = 3). (**H**) Multilayer histogram showing the quantified amount of surface HA of MCF-7 control (empty vector), single overexpression (HAS2, HYAL1), and double overexpressing (HAS2 and HYAL1) cells measured via flow cytometry using biotinylated HA-binding protein and Cy5/FITC-conjugated Streptavidin. One representative experiment is shown (*n* = 3). (**I**) Representative Western blot (*n* = 2) result of protein lysates (20 µg) of MCF-7 control (empty vector), single overexpression (HAS2, HYAL1) and double overexpressing (HAS2 and HYAL1) cells showing expression of different HA receptors, CD44 and RHAMM. β-Actin was used as loading control. Statistical significance was determined using unpaired two-tailed Student’s *t*-tests. The assumption of homogeneity of variance was tested using Levene’s Test of Equality of Variances (*p* > 0.05). Values are means +/− s.d. * *p* < 0.05.

Figure 6A,B show the successful HAS2 or/and HYAL1 overexpression in single (HAS2 and HYAL1) and double overexpressing (HAS2 and HYAL1) cells via qRT-PCR. MCF-7 HAS2 cells showed approximately 1200-fold higher HAS2 mRNA levels than the corresponding control cells (empty vector). In contrast, the double overexpressing variant (HAS2 and HYAL1) showed a 400-fold higher HAS2 mRNA level than the control. In both the single (HYAL1) and double overexpressing (HAS2 and HYAL1) cells, an approximately 12- to 16-fold increase in HYAL1 mRNA expression level compared to the control was detected.

In addition to the increased mRNA level, the functionality of both enzymes was tested. For the HYAL1-activity gel assay (Figure 6C), HMW-HA molecules were treated with cell lysates from the aforementioned cell lines, prior to loading in the agarose gel. Here, HA fragments are separated based on their molecular size. If cellular HYAL1 is active, HMW-HA can be cleaved into LMW-HA fragments that diffuse faster in the agarose gel, and can be detected (blue staining) in the lower half of the gel. HMW-HA, in contrast, remains in the upper half of the gel. A functional HYAL1 enzyme could be proven in the single HYAL1-overexpressing cells as well in the HAS2/HYAL1-overexpressing MCF-7 cells, with an even more pronounced enzymatic activity in the latter. Using a HA-ELISA system, we could further demonstrate the functionality of HAS2 (Figure 6D). Here, the synthesized and secreted amount of total HA, as well as LMW-HA, was measured. In comparison with the low endogenous HA secreted levels of MCF-7 cells (Figure 1C), the control cells (empty vector) as well as the single HYAL1-overexpressing MCF-7 cells, secreted approximately 10–50 ng HA/10^6^ cells. After HAS2 overexpression, alone or in combination with HYAL1, approximately 200–300 ng HA/10^6^ cells were measured, which is a level comparable to the one observed in the TNBC cell lines (Figure 1C). Considering the amount of LMW-HA, and, again, proving the functionality of HYAL1, both HYAL1-overexpressing MCF-7 sublines generated more LMW-HA compared to control cells, although these differences were not statistically significant. The increased amount of HA in HAS2-overexpressing and double overexpressing (HAS2 and HYAL1) cells could, additionally, be visualized via histochemical staining with a specific HA-binding protein (Figure 6E), in contrast with the lack of staining in control cells and HYAL1-overexpressing MCF-7 cells. 

After successfully confirming the overexpression and functionality of HAS2 and HYAL1 in MCF-7 cells, the impact of these enzymes on the BBB was analyzed as described in the previous sections. Contrary to our expectations, HAS2- and/or HYAL1-overexpressing MCF-7 cells showed neither altered adhesion to hBMECs (Figure 6F) nor an increased invasion ability through the endothelial monolayer (Figure 6G) compared to the control cells. In order to understand the cause of these unexpected results, the amount of surface-bound HA, the so-called pericellular HA-coat, was further quantified via FACS (Figure 6H). Here, as expected, low HA levels, namely, approx. 6% HA-cell positivity, were detected in the control and HYAL1-overexpressing MCF-7 cells. Interestingly, although the HAS2-overexpressing MCF7 cells secreted comparable HA levels to the TNBC cells, as previously demonstrated via HA-ELISA, the number of cells displaying pericellular HA staining was remarkably lower: 30–38% and 80% for HAS2-overexpressing MCF-7 and TNBC cells, respectively (Figure 1E). These findings led us to assume that HA2-overexpressing MCF-7 cells produce the same amount of HA as TNBCs, but cannot bind the synthesized HA to the cell surface to the same extent. Based on this, the expression of two HA receptors, CD44 (80 kDa) and RHAMM (85 kDa), was analyzed in the overexpressing cells as well as in the native BC cell lines using Western blot (Figure 6I). A strong CD44 expression was detected in both TNBC cell lines (MDA-MB-231, MDA-MB-231-BR), whereas MCF-7 cells, as well as the HAS2 and/or HYAL1-overexpressing sublines, did not express CD44. Regarding the expression of RHAMM, a HA receptor mainly responsible for signaling, higher expression levels were found in luminal MCF-7 cells than in TNBC cells (Supplementary Figure S3A). The overexpression of HAS2 in MCF-7 cells led to a down-regulation of RHAMM expression, whereas an HYAL1 overexpression led to a slight up-regulation of RHAMM. Both effects seemed to be mutually annulled, as HAS2- and HYAL1- overexpressing cells showed no strong difference in RHAMM expression as compared to control cells (Supplementary Figure S3B).

### 3.8. CD44 Knockdown in MDA-MB-231-BR Does Not Affect HA Metabolism but Reduces the Pericellular HA Coat 

**Figure 7:**
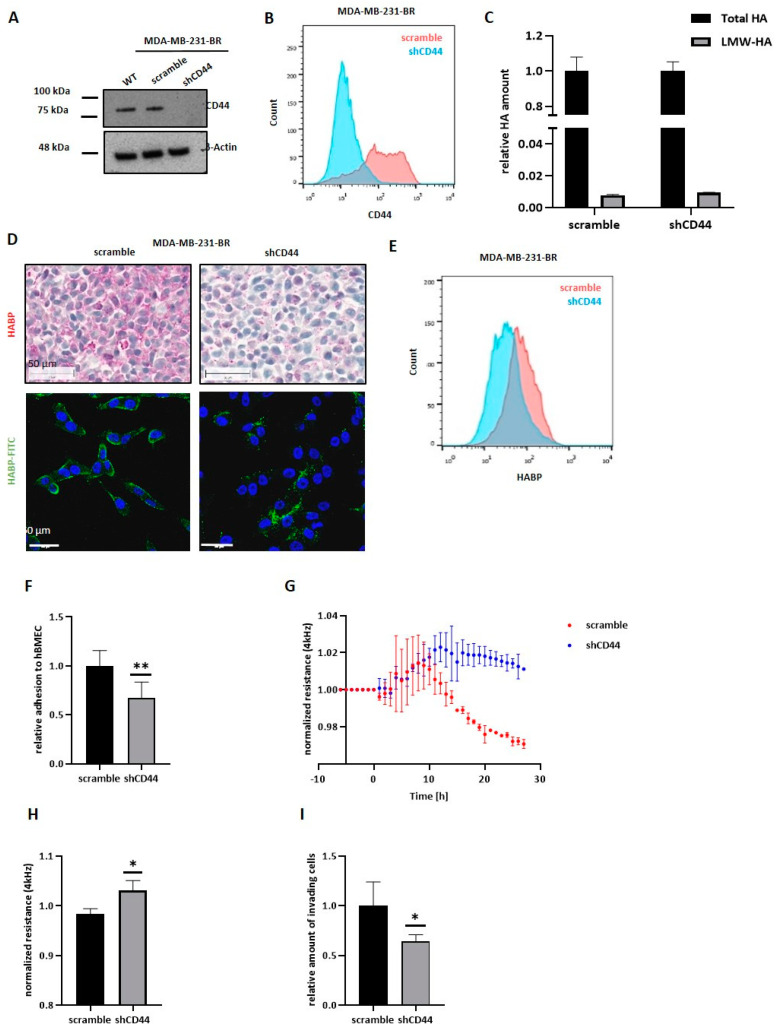
CD44 knockdown in MDA-MB-231-BR and its impact on HA metabolism and interaction with the BBB. (**A**) Representative Western blot (*n* = 2) result of protein lysates (20 µg) of MDA-MB-231-BR WT, control (scramble), and CD44 KD (shCD44) showing expression of the HA receptor CD44. β-Actin was used as a loading control. (**B**) Histogram showing the quantified amount of CD44 measured via flow cytometry. One representative experiment is shown (*n* = 2) (**C**) Bar graph displaying ELISA results on secreted total HA and LMW-HA levels (ng/10^6^ cells) of MDA-MB-231-BR control (scramble) and CD44 KD (shCD44) cells. Cell supernatant was collected and used for ELISA (representative experiment, *n* = 3). (**D**) Histochemical staining of cells in agar for HA using biotinylated HA binding protein, scale corresponds to 50 µm. Fluorescence staining of nuclei (DAPI, blue) and HA (FITC, green) using biotinylated HABP and FITC-conjugated Streptavidin. Scale bars represent 50 µm. (**E**) Histogram showing the quantified amount of surface HA measured via flow cytometry. One representative experiment is shown (*n* = 2). (**F**) The adhesion ability of MDA-MB-231-BR control (scramble) and CD44 knockdown (shCD44) cells to adhere to primary human brain endothelial cells (hBMECs) was analyzed under static conditions. The relative amount (to scramble = 1) of adhesive cells is shown (*n* = 3). (**G**) ECIS: normalized resistance at 4 kHz (values were set as 1 prior treatment; representative experiment; *n* = 3) (**H**) Bar graphs displaying relative resistance values at 4 kHz under the influence of different tumor cells (*n* = 3). (**I**) Invasion potential of MDA-MB-231-BR control (scramble) and CD44 knockdown (shCD44) cells through hBMECs measured by the Transwell assay (*n* = 3). Statistical significance was determined using unpaired two-tailed Student’s *t*-tests. The assumption of homogeneity of variance was tested using Levene’s Test of Equality of Variances (*p* > 0.05). Values are means +/− s.d. * *p* < 0.05, ** *p* < 0.005.

The previous results suggest a strong dependence on CD44 expression for the generation of a pericellular HA-coat in BC cells, which, in turn, was essential for the impact of HYAL1 on the BBB. Based on this observation, next we performed CD44 down-regulation via specific shRNA in MDA-MB-231-BR cells, which could be corroborated via Western blot and FACs analysis in the corresponding shCD44 subline (Figure 7A,B, quantification of protein expression in Supplementary Figure S3C). Besides the standard CD44 variant (CD44_S), the human CD44 exists in several isoforms (v1 to v10), due to alternative splicing from exon 6 to exon 15. QRT-PCR analysis considering these 10 variable exons (v-exon) showed a down-regulation not only of the CD44 standard variant, but of all variable exons targeted by the specific shRNA sequence in the shCD44 subline as compared with the control cells (Supplementary Figure S3D). The impact of CD44 knockdown (KD) on the HA metabolism was subsequently analyzed by HA-ELISA in the aforementioned MDA-MB-231-BR subline (Figure 7C). The relative amount of LMW-HA compared to the total HA amount is shown for both cell lines. Neither the total secreted amount of HA nor the amount of LMW-HA differed between scramble and shCD44 cells, indicating that lower expression levels of the HA receptor CD44 do not affect the HA metabolism. However, CD44 down-regulation led to a strong reduction in the pericellular HA-coat as visualized using histochemical and fluorescence staining with a specific HA-binding protein (Figure 7D). Here, generally less detected HA, and especially less surface-bound HA for MDA-MB-231-BR shCD44 compared to scramble cells, could be detected. Reduced surface-bound HA in shCD44 cells was also confirmed by FACS analysis (Figure 7E). 

### 3.9. CD44 Knockdown in MDA-MB-231-BR Leads to a Less Metastatic Phenotype In Vitro 

The impact of CD44 KD on the interaction of MDA-MB-231-BR cells with the brain endothelium was subsequently analyzed. Here, a reduced CD44 expression led to significantly less adhesion (32% less adhesion) to brain endothelial cells compared to the control cells (Figure 7F), with a relative number of adherent tumor cells (shCD44) to the brain endothelium similar to those of the HYAL1 KO cells (as shown in Figure 5A). Furthermore, the influence of the CD44 KD on the integrity of the BBB was measured by ECIS at 4 kHz. Figure 7G shows the time course of a representative experiment, in which impedance values were set as one, 10 min before the addition of tumor cells. After tumor cell addition, we observed an increase in the impedance value in both cases (scramble and shCD44). For the control group, however, this increase was only noticeable for a short time (about 10 min), and then transformed into a strong reduction in resistance, as expected. In contrast, after the addition of shCD44 cells, the resistance value remained constant. Graph bars in Figure 7H represent a summary of three independent experiments. Moreover, we observed a strong and significant reduction in cell invasion through the brain endothelium in CD44 KD cells compared with the corresponding control cells (Figure 7I). 

## 4. Discussion

BC patients with BM suffer from reduced life quality and expectancy. In this context, many efforts have been made to identify primary BC patients with a high risk of developing BM. Screening options should be examined in future studies, as these patients represent an important group for preventive strategies. Although certain BC molecular subtypes exhibit a higher predisposition to development of BM, little is known about the specific molecular players required for a tumor cell to successfully overcome the BBB and colonize in the brain. We could previously show a significant association between the expression of the synthase HAS2 and the hyaluronidase HYAL1 in the primary tumor of BC patients and the development of BM [26]. While Has-2 is responsible for the synthesis of HA polymers, Hyal-1 cleaves these into low molecular weight fragments, which have been described to exert alternative functions. In this study, we have explored the relevance of the HA metabolism and, more precisely, of the LMW-HA fragments, on the ability of BC cells to pass the BBB and develop cerebral metastasis. We found that Hyal-1 and, specifically, the Hyal-1-generated LMW-HA fragments, are essential for the formation of a functional pericellular HA-coat, which in turn promotes the tumor cell interaction with the brain endothelium and supports BCBM development.

Several studies have previously demonstrated the association of hyaluronan with more aggressive phenotypes in cancer, including BC, being recently included in the “hallmarks of cancer” [33]. In line with these findings, our present results demonstrate a higher HA metabolism rate in the more aggressive TNBC cell line, MDA-MB-231, and its brain-seeking subline, MDA-MB-231-BR, compared to the luminal BC cell line MCF-7. Here, a robust pericellular HA-coat was detected in the TNBC cell lines, with the brain-seeking subline displaying a more expressed surface HA deposit. Remarkably, although both TNBC cell lines displayed a strong ability to adhere to the brain endothelial monolayer and invade in vitro, only the brain-seeking BC cell line was dependent on its membrane-associated HA, as shown after pericellular HA-removal with exogenous hyaluronidases. Similarly, the reduction in the surface-associated endothelial HA via hyaluronidase treatment resulted in decreased adhesion and invasion ability of brain-metastatic BC cells, but not of the parental cell line, underlining the key role of hyaluronan for the development of BM. To our knowledge, this is the first report on the role of pericellular HA for adhesion and the invasion of BC cells through the BBB in vitro. An association between the presence of a cellular HA-coat and increased cell adhesion, motility, and invasive potential has been previously described in several tumor entities, including BC [23]. A study by Brett et al. showed that the enzymatic removal of HA significantly inhibits cancer cell extravasation and invasion through human umbilical vein endothelial cells (HUVECs) [34]. These data and our own suggest that membrane-bound HA represents a docking structure that mediates tumor cell adhesion to the endothelial layer and, additionally, triggers pro-invasive signaling. Although the parental and the brain-seeking subline synthesize approximately the same amount of HA, the flow cytometry and HA-fluorescence staining analyses showed higher levels of HA and a more compact staining pattern on the surface of MDA-MB-231-BR cells in comparison to the parental cell line. This result indicates a more pronounced and functional HA-coat in the brain-seeking cells. One explanation might be the presence of HA fragments of different sizes in the two TNBC cell lines. It is known that large HA fragments, so-called HMW-HA, which are mainly present under physiological conditions, exert anti-angiogenic, anti-inflammatory, and anti-proliferative functions, whereas smaller fragments or LMW-HA, which are, rather, found under pathological conditions, activate signaling cascades that promote angiogenesis, immune cell influx proliferation, and migration [21,22]. As mentioned before, we have previously demonstrated that high tumoral Hyal-1 expression levels in the primary tumor significantly correlate with BM development in BC patients [26]. Taking these data into consideration, we hypothesized that the brain-seeking cell line preferentially synthetizes LMW-HA, which might be responsible for the formation of a compact and functional HA-coat, thereby enhancing tumor cell adhesion to the endothelium and invasion through the BBB. However, there are no methods currently available to discriminate between the HMW-HA and LMW-HA structures at the cell membrane. The HA-binding protein used to visualize and quantify HA via fluorescence staining, which is also used in flow cytometry analysis, recognizes and binds both structures and provides information about the total HA amount. Therefore, in order to explore the specific role of LMW-HA, we generated an HYAL1 knockout in the TNBC brain seeking subline. As expected, both HYAL1 KO clones were confirmed to secrete less LMW-HA, while the total amount of secreted HA was not altered. Indeed, at the cell surface, the amount of total bound HA measured by flow cytometry was similar for all KO clones and the corresponding control cells. This means that, despite HYAL1 KO, these cells were able to generate a pericellular HA-coat. HA fluorescence staining, however, revealed a remarkably reduced and thinner HA coat in the HYAL1 KO clones, indicating that LMW-HA is essential, but is not the only component of the pericellular HA-coat. A study conducted by Reiprich et al. recently showed the formation of a thicker pericellular HA coat after HAS3 overexpression when compared with HAS2 [35]. Thus, the smaller HA polymers synthesized by Has-3 appear to form a thicker coat compared to the larger Has-2-generated HA polymers. 

Despite the not yet fully elucidated composition of the pericellular HA coat on tumor cells, and, in particular, the specific role of Hyal-1-synthesized LMW-HA fragments in this coat, we could demonstrate a significant impact of Hyal-1 on the development of BM in vitro as well as in vivo. In all three major steps involved in the brain-metastatic process, namely adhesion, disruption of the BBB, and invasion, HYAL1 KO cells showed a less aggressive phenotype and reduced metastasis-promoting properties. The reduced adhesiveness to the brain endothelium argues for a crucial mechanical role of LMW-HA fragments. On the other hand, aberrant signaling also occurs due to the lower amount of LMW-HA, likely resulting in reduced BBB integrity and invasion through the BBB. These effects were confirmed in vivo using a mouse model with intracardiac injection. For the HYAL1 KO clone, significantly reduced brain metastasis was detected, either by BLI signals within the brains, or macroscopically by histochemical HA-BP staining in mice injected with HYAL1 KO, compared to the control group. 

Particularly striking were the morphological alterations observed in MDA-MB-231-BR cells with HYAL1 KO, which displayed a more epithelial phenotype. These newly acquired cellular characteristics might also explain the significantly reduced adhesion and invasion related to the BBB observed in these cells. A previous study by Zoltan-Jones et al. showed that HA can promote cell invasiveness and EMT; more precisely, overexpression of HAS2 in the epithelial BC cell line MCF10A promoted both invasiveness and EMT [36]. In contrast, in the present work, the HYAL1 depletion and, in turn, the reduction of LMW-HA fragments in the brain-seeking TNBC cell line did not affect MET. This observation allowed us to assume that high expression levels of Has-2 and, therefore, high HMW-HA production promotes a mesenchymal phenotype in tumor cells, and is independent of the amount of LMW-HA. Moreover, we found that HA also affected the cytoskeleton organization, likely through the binding to its cognate receptor CD44. To date, a modulating role of HYAL2, but not HYAL1, within the ERM-related cytoskeletal interactions has been shown only in fibroblasts [37]. However, future experiments are required to elucidate the mechanism underlying the observed changes in the actin cytoskeleton in HYAL1 depleted cells.

To our surprise, when we overexpressed HAS2 and HYAL1 in the luminal BC cell line MCF7, neither increased tumor cell adhesion, nor migration through the brain endothelium, nor any morphological alteration was observed. MCF-7 overexpressing cells synthesized and secreted a similar amount of LMW-HA compared to MDA-MB-231-BR cells, but their pericellular HA-coat was considerably weaker, suggesting that LMW-HA fragments might only have an impact on the BBB as a part of the pericellular coat, and not in their secreted and soluble form. Here, a possible explanation for the lack of a proper HA-coat, even in the presence of sufficient HA molecules, might be the absence of the HA receptor CD44 in MCF-7 cells. Indeed, we could further confirm the key role of CD44 in the formation of the HA-coat in MDA-MB-231-BR cells. Here, a less pronounced HA-coat was detected in CD44 knockdown cells in comparison to the control cells. Moreover, down-regulation of CD44 in MDA-MB-231-BR resulted in a reduced HA-coat. This points to a key role of CD44 as a mediator for the formation of a functional HA-coat, rather than during HA-internalization and subsequent synthesis of LMW-HA.

Taken together, the HA metabolism plays a key role in the development of BM in BC. Precisely, low molecular weight HA, mainly generated by the hyaluronidase Hyal1, supports the formation of a pericellular HA coat as well as pro-metastatic signaling in BC cells. Both effects are dependent on the binding of LMW-HA to its receptor, CD44, and ultimately promote BM development. In this context, inhibition of the interaction between Hyal-1 generated LMW-HA fragments and CD44 might represent a target for future therapeutic interventions. 

## 5. Conclusions

Due to an existing lack of effective treatment, BCBM is becoming a critical issue. Due to the semi-permeable BBB, the passage of therapeutic agents into the brain is difficult, and effective treatment is generally not possible. In order to enhance options and success of treatment, an improved understanding of the molecular mechanisms of how tumor cells pass the BBB and, thus, develop metastatic lesions is required. In the present work, the precise and outstanding role of Hyal-1 during BCBM development was revealed for the first time. The down-regulation of the hyaluronidase Hyal-1 in the brain-seeking BC cell line MDA-MB-231-BR, and the associated reduced amount of LMW-HA, significantly reduced the essential steps during brain metastasis in vitro, as well as the number of brain metastases in vivo. In this regard, LMW-HA appears to be responsible for the formation of a pericellular HA-coat around tumor cells, and interacts with brain endothelium. A prerequisite for the HA-coat-mediated impact on the metastatic behavior of BC cells is the presence of the HA receptor CD44. Aside from its essential function in the context of the HA metabolism, and, thus, the synthesis of LMW-HA fragments, CD44 plays a key role in the formation of the pericellular HA coat on brain metastatic BC cells. By inhibiting the interaction with the brain endothelium, tumor cells could lose the prerequisite for the formation of brain metastases by inhibiting the “signaling” of the LMW-HA-CD44 axis, and, thus, the formation of a pericellular HA coat. 

## Data Availability

The datasets used and/or analyzed during the current study are available from the corresponding author upon reasonable request.

## Supplementary Materials

The following supporting information can be downloaded at: https://www.mdpi.com/article/10.3390/cells11203275/s1, Figure S1: Expression of HA metabolic enzymes and quantification of CTCs in HYAL1 KO clones; Figure S2: Expression of CD44 variants in HYAL1 KO clones. Figure S3: Quantification of protein expression and expression of CD44 variants in MDA-MB-231-BR CD44 knockdown cells as well as MCF-7 overexpressing cells. Reference [38] is cited in the Supplementary Materials.

## References

[1] Tabouret E., Chinot O., Metellus P., Tallet A., Viens P., Gonçalves A. (2012). Recent trends in epidemiology of brain metastases: An overview. Anticancer Res..

[2] Saha A., Ghosh S.K., Roy C., Choudhury K.B., Chakrabarty B., Sarkar R. (2013). Demographic and clinical profile of patients with brain metastases: A retrospective study. Asian J. Neurosurg..

[3] Klos K.J., O’Neill B.P. (2004). Brain metastases. Neurologist.

[4] Quigley M.R., Fukui O., Chew B., Bhatia S., Karlovits S. (2013). The shifting landscape of metastatic breast cancer to the CNS. Neurosurg. Rev..

[5] Lee S.S., Ahn J.-H., Kim M.K., Sym S.J., Gong G., Ahn S.D., Kim S.-B., Kim W.K. (2008). Brain metastases in breast cancer: Prognostic factors and management. Breast Cancer Res. Treat..

[6] Ogawa K., Yoshii Y., Nishimaki T., Tamaki N., Miyaguni T., Tsuchida Y., Kamada Y., Toita T., Kakinohana Y., Tamaki W. (2008). Treatment and prognosis of brain metastases from breast cancer. J. Neuro-Oncol..

[7] Sperduto P.W., Kased N., Roberge D., Xu Z., Shanley R., Luo X., Sneed P.K., Chao S.T., Weil R.J., Suh J. (2012). Effect of tumor subtype on survival and the graded prognostic assessment for patients with breast cancer and brain metastases. Int. J. Radiat. Oncol. Biol. Phys..

[8] Niwińska A., Pogoda K., Murawska M., Niwiński P. (2011). Factors influencing survival in patients with breast cancer and single or solitary brain metastasis. Eur. J. Surg. Oncol..

[9] Gil-Gil M.J., Martinez-Garcia M., Sierra A., Conesa G., del Barco S., González-Jimenez S., Villà S. (2014). Breast cancer brain metastases: A review of the literature and a current multidisciplinary management guideline. Clin. Transl. Oncol..

[10] Fortin D. (2012). The blood-brain barrier: Its influence in the treatment of brain tumors metastases. Curr. Cancer Drug Targets.

[11] Ballabh P., Braun A., Nedergaard M. (2004). The blood-brain barrier: An overview: Structure, regulation, and clinical implications. Neurobiol. Dis..

[12] Abbott N.J., Patabendige A.A., Dolman D.E., Yusof S.R., Begley D.J. (2010). Structure and function of the blood-brain barrier. Neurobiol. Dis..

[13] Abbott N.J. (2013). Blood-brain barrier structure and function and the challenges for CNS drug delivery. J. Inherit. Metab. Dis..

[14] Kennecke H., Yerushalmi R., Woods R., Cheang M.C.U., Voduc D., Speers C.H., Nielsen T.O., Gelmon K. (2010). Metastatic behavior of breast cancer subtypes. J. Clin. Oncol..

[15] Yau T., Swanton C., Chua S., Sue A., Walsh G., Rostom A., Johnston S.R., O’Brien M.E.R., Smith I.E. (2006). Incidence, pattern and timing of brain metastases among patients with advanced breast cancer treated with trastuzumab. Acta Oncol..

[16] Witzel I., Kantelhardt E.J., Milde-Langosch K., Ihnen M., Zeitz J., Harbeck N., Jänicke F., Müller V. (2011). Management of patients with brain metastases receiving trastuzumab treatment for metastatic breast cancer. Onkologie.

[17] Witzel I., Oliveira-Ferrer L., Pantel K., Müller V., Wikman H. (2016). Breast cancer brain metastases: Biology and new clinical perspectives. Breast Cancer Res..

[18] Jeon W., Jang B.-S., Jeon S.H., Kim J.H., Kim Y.J., Kim S.H., Kim C.-Y., Han J.H., Kim I.A. (2018). Analysis of survival outcomes based on molecular subtypes in breast cancer brain metastases: A single institutional cohort. Breast J..

[19] Witzel I., Laakmann E., Weide R., Neunhöffer T., Park-Simon T.-J., Schmidt M., Fasching P., Hesse T., Polasik A., Mohrmann S. (2018). Treatment and outcomes of patients in the Brain Metastases in Breast Cancer Network Registry. Eur. J. Cancer.

[20] Fraser J.R., Laurent T.C., Laurent U.B. (1997). Hyaluronan: Its nature, distribution, functions and turnover. J. Intern. Med..

[21] Tian X., Azpurua J., Hine C., Vaidya A., Myakishev-Rempel M., Ablaeva J., Mao Z., Nevo E., Gorbunova V., Seluanov A. (2013). High-molecular-mass hyaluronan mediates the cancer resistance of the naked mole rat. Nature.

[22] Tavianatou A.G., Caon I., Franchi M., Piperigkou Z., Galesso D., Karamanos N.K. (2019). Hyaluronan: Molecular size-dependent signaling and biological functions in inflammation and cancer. FEBS J..

[23] Toole B.P. (2004). Hyaluronan: From extracellular glue to pericellular cue. Nat. Rev. Cancer.

[24] Heldin P., Delatorre M., Ytterberg D., Bergh J. (1996). Differential synthesis and binding of hyaluronan by human breast cancer cell lines. Oncol. Rep..

[25] Li P., Xiang T., Li H., Li Q., Yang B., Huang J., Zhang X., Shi Y., Tan J., Ren G. (2015). Hyaluronan synthase 2 overexpression is correlated with the tumorigenesis and metastasis of human breast cancer. Int. J. Clin. Exp. Pathol..

[26] Witzel I., Marx A.K., Müller V., Wikman H., Matschke J., Schumacher U., Stürken C., Prehm P., Laakmann E., Schmalfeldt B. (2017). Role of HYAL1 expression in primary breast cancer in the formation of brain metastases. Breast Cancer Res. Treat..

[27] Hamester F., Stürken C., Saygi C., Qi M., Legler K., Gorzelanny C., Robador J.R., Schmalfeldt B., Laakmann E., Müller V. (2022). Insights into the Steps of Breast Cancer-Brain Metastases Development: Tumor Cell Interactions with the Blood-Brain Barrier. Int. J. Mol. Sci..

[28] Ran F.A., Hsu P.D., Wright J., Agarwala V., Scott D.A., Zhang F. (2013). Genome engineering using the CRISPR-Cas9 system. Nat. Protoc..

[29] Oliveira-Ferrer L., Goswami R., Galatenko V., Ding Y., Eylmann K., Legler K., Kürti S., Schmalfeldt B., Milde-Langosch K. (2018). Prognostic Impact of CEACAM1 in Node-Negative Ovarian Cancer Patients. Dis. Markers.

[30] Pan C., Kumar C., Bohl S., Klingmüller U., Mann M. (2009). Comparative proteomic phenotyping of cell lines and primary cells to assess preservation of cell type-specific functions. Mol. Cell. Proteom..

[31] Alge C.S., Hauck S.M., Priglinger S.G., Kampik A., Ueffing M. (2006). Differential protein profiling of primary versus immortalized human RPE cells identifies expression patterns associated with cytoskeletal remodeling and cell survival. J. Proteome Res..

[32] Eigenmann D.E., Xue G., Kim K.S., Moses A.V., Hamburger M., Oufir M. (2013). Comparative study of four immortalized human brain capillary endothelial cell lines, hCMEC/D3, hBMEC, TY10, and BB19, and optimization of culture conditions, for an in vitro blood-brain barrier model for drug permeability studies. Fluids Barriers CNS.

[33] Caon I., Bartolini B., Parnigoni A., Caravà E., Moretto P., Viola M., Karousou E., Vigetti D., Passi A. (2020). Revisiting the hallmarks of cancer: The role of hyaluronan. Semin. Cancer Biol..

[34] Brett M.E., Bomberger H.E., Doak G.R., Price M.A., McCarthy J.B., Wood D.K. (2018). In vitro elucidation of the role of pericellular matrix in metastatic extravasation and invasion of breast carcinoma cells. Integr. Biol..

[35] Reiprich S., Hofbauer E., Kiderlen S., Clausen-Schaumann H., Böcker W., Aszódi A., Schönitzer V. (2020). Adhesive Properties of the Hyaluronan Pericellular Coat in Hyaluronan Synthases Overexpressing Mesenchymal Stem Cells. Int. J. Mol. Sci..

[36] Zoltan-Jones A., Huang L., Ghatak S., Toole B.P. (2003). Elevated hyaluronan production induces mesenchymal and transformed properties in epithelial cells. J. Biol. Chem..

[37] Duterme C., Mertens-Strijthagen J., Tammi M., Flamion B. (2009). Two novel functions of hyaluronidase-2 (Hyal2) are formation of the glycocalyx and control of CD44-ERM interactions. J. Biol. Chem..

[38] Nehmann N., Wicklein D., Schumacher U., Müller R. (2010). Comparison of two techniques for the screening of human tumor cells in mouse blood: Quantitative real-time polymerase chain reaction (qRT-PCR) versus laser scanning cytometry (LSC). Acta Histochem..

