# Supplementary material for: Key Role of Hyaluronan Metabolism for the Development of Brain Metastases in Triple-Negative Breast Cancer
Source: Cells. 2022 Oct 18;11(20):3275. doi: 10.3390/cells11203275 (PMC9600690; doi:10.3390/cells11203275)
Supplement: Supplementary file 1 [file cells-11-03275-s001.zip › Supplementary Figure S1.pdf]

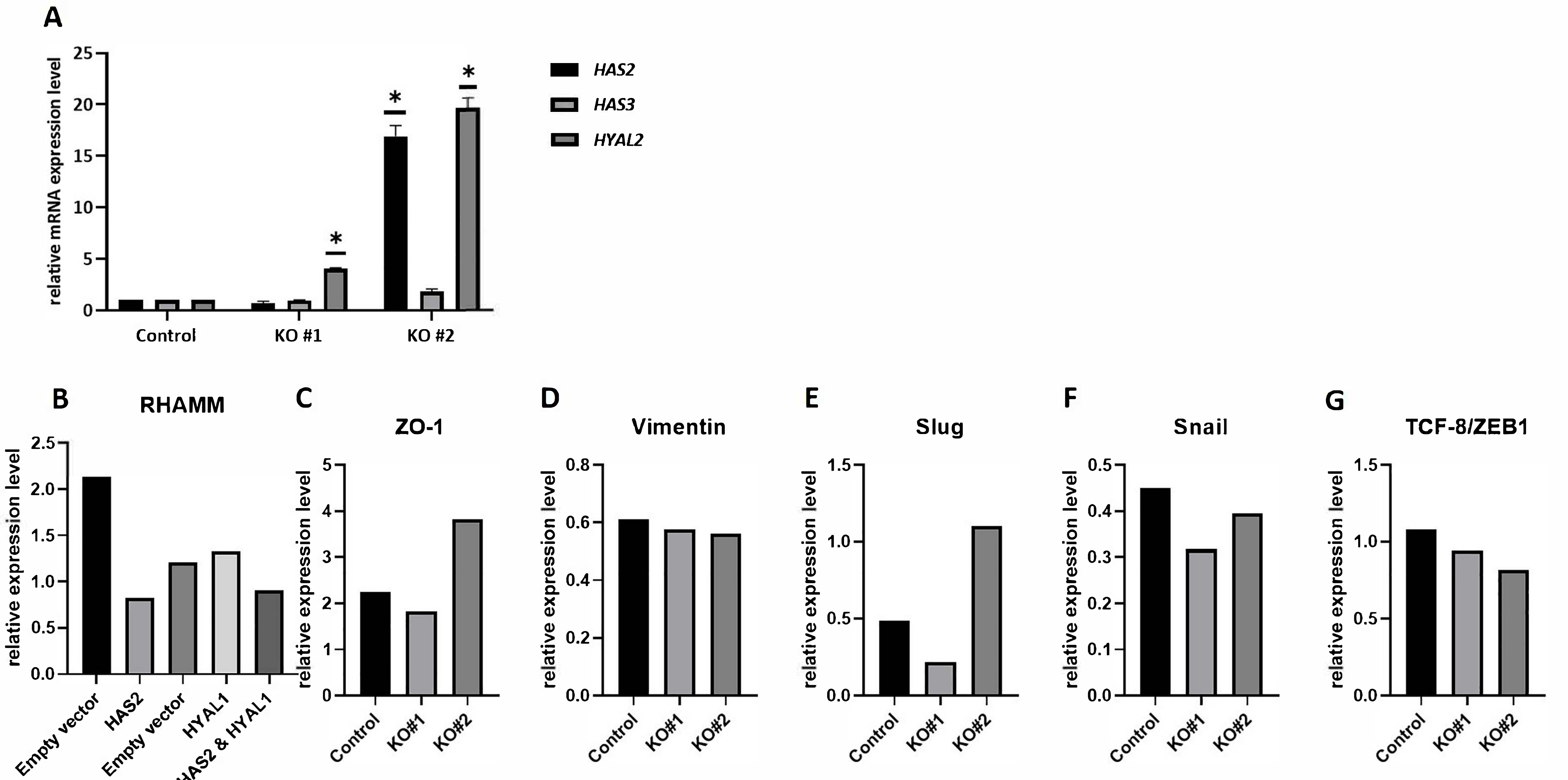

Supplementary Figure S1: Expression of HA metabolic enzymes and quantification of different EMT protein expression levels in HYAL1 KO clones

(A) Relative mRNA expression level of HAS2, HAS3, and HYAL2 measured by qRT-PCR. Values are normalized to corresponding GAPDH expression (n=2). Relative EMT protein expression from representative Western Blot was quantified by densitometry of **(B)** β-Catenin, **(C)** ZO-1, **(D)** Vimentin, **(E)** Slug, **(F)** Snail and **(G)** TCF-8/ZEB1 for MDA-MB-231-BR Control as well as HYAL1 KO #1 and KO #2 cells. Statistical significance was determined using unpaired two-tailed Student´s t-tests. The assumption of homogeneity of variance was tested using Levene´s Test of Equality of Variances ($p>0.05$). Values are means + s.d. *$p<0.05$.
