# Supplementary material for: Key Role of Hyaluronan Metabolism for the Development of Brain Metastases in Triple-Negative Breast Cancer
Source: Cells. 2022 Oct 18;11(20):3275. doi: 10.3390/cells11203275 (PMC9600690; doi:10.3390/cells11203275)
Supplement: Supplementary file 1 [file cells-11-03275-s001.zip › Supplementary Figure S2.pdf]

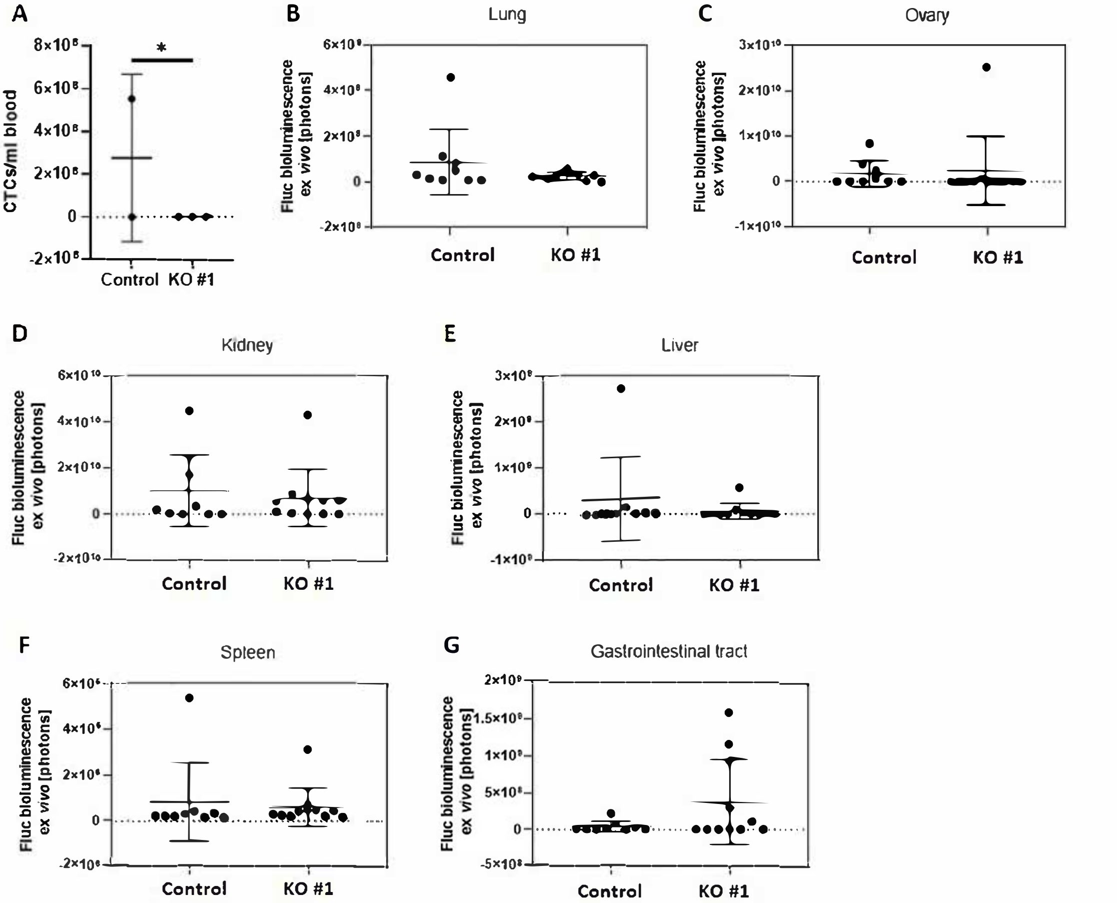

Supplementary Figure S2: Additional in vivo data of HYAL1 KO mouse model.
(A) Number of CTCs quantified by ALU-PCR from blood. Ex vivo BLI signals from **(B)** lungs, **(C)** ovary, **(D)** kidney, **(E)** liver, **(F)** spleen and **(G)** gastrointestinal tract of both groups (MDA-MB-231-BR control: n=9, HYAL1 KO #1: n=11). Statistical significance was determined using unpaired two-tailed Student´s t-tests. The assumption of homogeneity of variance was tested using Levene´s Test of Equality of Variances (p>0.05). Values are means + s.d. *p<0.05.
