# Supplementary material for: Key Role of Hyaluronan Metabolism for the Development of Brain Metastases in Triple-Negative Breast Cancer
Source: Cells. 2022 Oct 18;11(20):3275. doi: 10.3390/cells11203275 (PMC9600690; doi:10.3390/cells11203275)
Supplement: Supplementary file 1 [file cells-11-03275-s001.zip › Supplementary Figure S3.pdf]

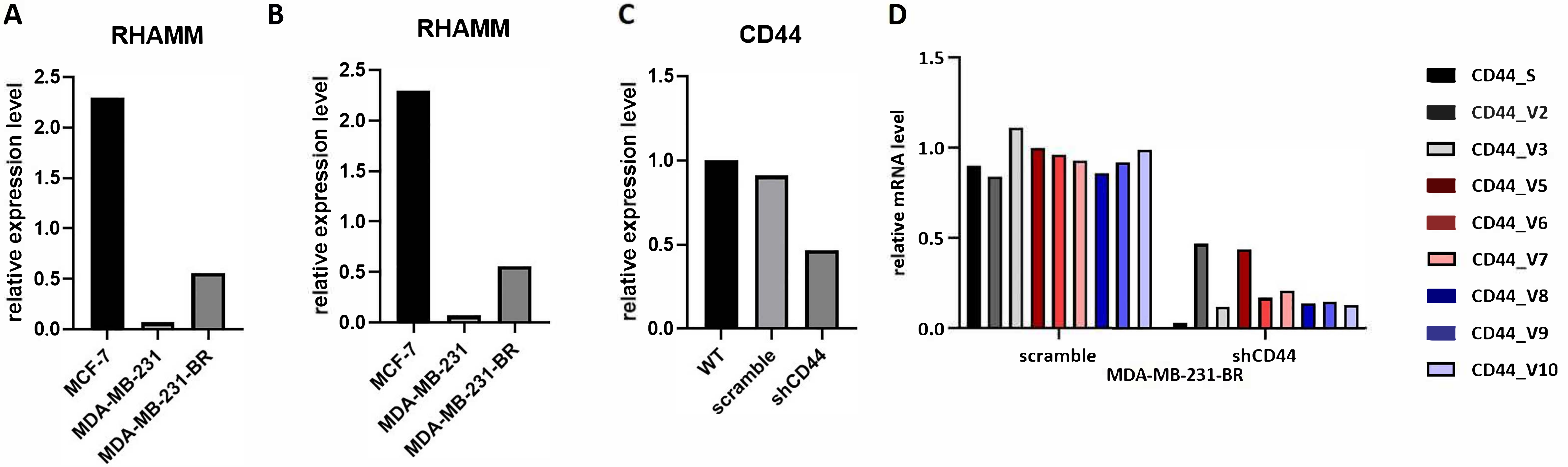

Supplementary Figure S3: Quantification of protein expression and expression of CD44 variants in MDA-MB-231-BR CD44 knockdown cells as well as MCF-7 overexpressing cells

(A) Relative protein expression from representative Western Blot was quantified by densitometry of RHAMM for MCF-7, MDA-MB-231 and MDA-MB-231-BR cells. **(B)** Relative protein expression from representative Western Blot was quantified by densitometry of RHAMM for MCF-7 overexpressing cells. Expression level of empty vector was set as 1. **(C)** Relative CD44 mRNA level of CD44 standard (CD44_S) and variable exons (CD44_V1-10) in MDA-MB-231-BR control (scramble) and CD44 KD (shCD44) measured by qRT-PCR. Values are normalized to corresponding GAPDH expression. **(D)** Relative protein expression from representative Western Blot was quantified by densitometry of CD44 from MDA-MB-231-BR control cells as well as CD44 knockdown cells. Expression level of MDA-MB-231-BR WT cells was set as 1. Statistical significance was determined using unpaired two-tailed Student´s t-tests. The assumption of homogeneity of variance was tested using Levene´s Test of Equality of Variances ($p>0.05$). Values are means ± s.d.
