# Supplementary material for: Key Role of Hyaluronan Metabolism for the Development of Brain Metastases in Triple-Negative Breast Cancer
Source: Cells. 2022 Oct 18;11(20):3275. doi: 10.3390/cells11203275 (PMC9600690; doi:10.3390/cells11203275)
Supplement: Supplementary file 1 [file cells-11-03275-s001.zip › Supplementary Materials and Methods_final.pdf]

CRISPR/Cas9-HYAL1 knockout in MDA-MB-231-BR

The guide RNAs for the HYAL1 knockout (KO) were generated using the CRISPR guide RNA design tool *guide scan*. The following two guide sequences were used:

1. **guide #1** (score:27, off-targets:0, Exon2, coordinates: chr3:50302887-50302909)
   Guide sequence: CGATATGGCCCAAGGCTTTA NGG
   Guide1: 5′CACC CGATATGGCCCAAGGCTTTA 3′
   Complementary: 3′GCTATACCGGGTTCCGAAATCAAA 5′
2. **guide #2** (score:61, off-targets:0, Exon2, coordinates: chr3:50302895-50302917)
   Guide sequence: ACCTTACTCGATATGGCCCA NGG
   Guide1: 5′CACC ACCTTACTCGATATGGCCCA 3′
   Complementary: 3′TGGAATGAGCTATACCGGGTCAAA 5′

For further cloning, the sgRNA oligos named HYAL1_e2_g1 and HYAL1_e2_g2 were designed: HYAL1_e2_g1 (FP: CACCCGATATGGCCCAAGGCTTTA; RP: AAACTAAAGCCTTGGGCCATATCG) HYAL1_e2_g2 (FP: CACCACCTTACTCGATATGGCCCA; RP: AAACTGGGCCATATCGAGTAAGGT)

Genomic DNA isolation and Sanger sequencing

Isolation of genomic DNA was performed using QIAamp DNA Mini Kit (#51304, Qiagen, Hilden, Germany) according to manufacturer's instruction. For amplification of the genomic region targeted by the CRISPR/Cas9, PCR from genomic DNA of control cells (Ctrl) and HYAL1 KO cells and following primer was performed: HYAL1_seq_e2_g1/2 (FP CGGTGGATTAATGCGCAAGG; RP GTAGTAGG GGTAGGTGCCCA). Sanger sequencing analysis was done by TGCA Company, National Cancer Institute, Bethesda, Maryland, USA.

QRT-PCR primer sequences for CD44 splice variants

CD44_S (FP: AGTCACAGACCTGCCCAATGCCTTT; RP: TTTGCTCCACCTTCTTGACTCCCATG)
CD44_V2 (FP: GACAGCAACCAAGAGGCAAG; RP: TTTGTGTTGTTGTGTGAAGATGATT)
CD44_V3 (FP: CGTCTTCAAATACCATCTCAGCA; RP: ATCTTCATCATCAATGCCTGA)
CD44_V4 (FP: AACCACACCACGGGCTTT; RP: CATCCTTGTGGTTGTCTGAAGTA)
CD44_V5 (FP: ATGTAGACAGAAATGGCACCAC; RP: GTGCTTGTAGAATGTGGGGTC)
CD44_V6 (FP: GGCAACTCCTAGTAGTACACCG; RP: GTCTTCTCTGGGTGTTTGGC)
CD44_V7 (FP: CTCATACCAGCCATCCAATGC; RP: CTTCTTCCTGCTTGATGACCTC)
CD44_V8 (FP: CTCCAGTCATAGTATAACGCTTCA; RP: GTTGTCATTGAAAGAGGTCCTGT)
CD44_V9 (FP: AGCAGAGTAATTCTCAGAGCTTC; RP: CAGAGTAGAAGTTGTTGGATGGT),
CD44_V10 (FP: ATCATTCTGAAGGCTCAACTACTT; RP: TAAGGAACGATTGACATTAGAGTTG)

QRT-PCR primer sequences

Alu-PCR Primer (FP: TGGCTCACGCCTGTAATCCCA; RP: GCCACTACGCCCGGCTAATTT)
HAS1 (FP: GGCCTGGTACAACCAGAAGT; RP: CCTGGAGGTGTACTTGGTAGC)HAS2 (FP: TGGGGGAGATGTCCAGATTT; RP: CCAAAGCTACATTGGTTGCCC)
HAS3 (FP: TGGACTACATCCAGGTGTGC; RP: TGCACACAGCCAAAGTGGA)
HYAL1 (FP: CTGCTGGTGACCCCAATCT; RP: GAAGGGCCCCAGTGTAGTGT)
HYAL2 (FP: TCGTGGCTGACTCCCATA; RP: ACGCTGGCCCTTTAAGACTC)
GAPDH (FP: GTCAGTGGTGGACCTGACCT; RP: TGCTGTAGCCAAATTCGTTG)

Antibodies for Western Blot method

Detection	Blocking	1st Antibody	2nd Antibody
**β-Actin**	5 % MP for 1 h at RT	1:2000, in 1.5 % MP in TBS-T, overnight, 4 °C	α-rabbit, 1:8000 in 1,5 % MP in TBS-T,1 h RT
**ß-Catenin**	5 % MP for 1 h at RT and 3x 5 Min TBS-T washing	1:1000, in 5 % BSA in TBS-T, overnight, 4 °C	α-rabbit, 1:8000 in 1,5 % MP in TBS-T,1 h RT
**CD44**	5 % MP for 1 h at RT	1:200, in 1.5 % MP in TBS-T, overnight, 4 °C	α-mouse, 1:8000 in 1,5 % MP in TBS-T,1 h RT
**HSC-70**	5 % MP for 1 h at RT	1:100000, in 1.5 % MP in TBS-T, overnight, 4 °	α-mouse, 1:8000 in 1,5 % MP in TBS-T,1 h RT
**RHAMM**	5 % MP for 1 h at RT	1:100, in 1.5 % MP in TBS-T, overnight, 4 °C	α-mouse, 1:8000 in 1,5 % MP in TBS-T,1 h RT
**Slug**	5 % MP for 1 h at RT and 3x 5 Min TBS-T washing	1:1000, in 5 % BSA in TBS-T, overnight, 4 °C	α-rabbit, 1:8000 in 1,5 % MP in TBS-T,1 h RT
**Snail**	5 % MP for 1 h at RT and 3x 5 Min TBS-T washing	1:2000, in 5 % BSA in TBS-T, overnight, 4 °C	α-rabbit, 1:8000 in 1,5 % MP in TBS-T,1 h RT
**TCF8/ZEBa**	5 % MP for 1 h at RT and 3x 5 Min TBS-T washing	1:1000, in 5 % BSA in TBS-T, overnight, 4 °C	α-rabbit, 1:8000 in 1,5 % MP in TBS-T,1 h RT
**Vimentin**	5 % MP for 1 h at RT and 3x 5 Min TBS-T washing	1:2000, in 5 % BSA in TBS-T, overnight, 4 °C	α-rabbit, 1:8000 in 1,5 % MP in TBS-T,1 h RT
**ZO-1**	5 % MP for 1 h at RT and 3x 5 Min TBS-T washing	1:1000, in 5 % BSA in TBS-T, overnight, 4 °C	α-rabbit, 1:8000 in 1,5 % MP in TBS-T,1 h RT

TBS-T Buffer: 20 mM Tris-HCl (pH 7.6); Trizma®-Base –Solution (#77-86-1, Merck Millipore, Burlington, Massachusetts, USA), 0.137 M NaCl (#7647-14-5, Merck Millipore, Burlington, Massachusetts, USA), 10 % Tween-20 (#11332465001, Merck Millipore, Burlington, Massachusetts, USA)
MP: Milk powder (#1706404, Bio-Rad, Hercules, California, USA)
BSA: Bovine serum albumin (#8076.1, Carl Roth, Karlsruhe, Germany)

Embedding cells in paraffin for HABP histochemistry

Cells at a confluency of 80 % were first washed with 20 mL PBS (+/+) and then softly removed with a cell scraper. Cells were then centrifuged (5 min, 12000 rpm, RT) and fixed with 3.7 % phosphate-buffered formalin solution for 20 min at RT. After two washing steps, the cell pellet was finally suspended in 200 µL of 2 % liquid (liquefied in 55 °C water bath) Difco™ Agar Nobel (#11798223, Thermo Fisher Scientific, Waltham, Massachusetts, USA). The resulting cooled cell-agar pellet was embedded in paraffin. Here, the fixed cell pellet was dehydrated in an automated tissue processor and incubated in liquid paraffin in a pouring device twice for 5 h at 63 °C. The paraffin-impregnated cell pellet was transferred to metal molds with paraffin at 63 °C and cooled down.

Detection of circulating tumor cells via human Alu-PCR

DNA was isolated from 250 µL of whole blood using peqGOLD Blood & Tissue DNA Mini Kit (#13-3396-02, VWR International, Radnor, Pennsylvania, USA) according to the manufacturer´s protocol.

Afterward, quantitative real-time PCR for human Alu-sequences was performed as described previously [38]. Primer sequences can be found in supplementary materials and methods.
